# Covalent-Bridged Heterointerfaces via Grafted Triazine Organic Polymers Enable Directed Charge Transfer for Efficient Oxygen Reduction in Zn–Air Batteries

**DOI:** 10.1021/acsnano.5c11348

**Published:** 2025-08-25

**Authors:** Shan Chen, Jitao Shang, Fei-er Peng, Zihan Song, Yong Zheng, Yuhang Dai, Jiexin Zhu, Fei Guo, Xinliang Fu, Kaibin Chu, Xueying Cao, Yue Ouyang, Ivan P. Parkin, Yazhou Zhou, Guanjie He, Tianxi Liu, Wei Zong

**Affiliations:** † Hubei Key Laboratory of Pollutant Analysis & Reuse Technology, College of Chemistry and Chemical Engineering, 721339 Hubei Normal University, Huangshi 435002, P. R. China; ‡ Christopher Ingold Laboratory, Department of Chemistry, 4919 University College London, 20 Gordon Street, London WC1H 0AJ, U.K.; § Institute of Technological Sciences, 12390 Wuhan University, Wuhan, Hubei 430072 P. R. China; ∥ Department of Engineering Science, 6396 University of Oxford, Parks Road, Oxford OX1 3PJ, U.K.; ⊥ College of Materials and Chemical Engineering, Key Laboratory of Inorganic Nonmetallic Crystalline and Energy Conversion Materials, 26476 China Three Gorges University, Yichang 443002, P. R. China; # College of Materials Science and Engineering, 165082 Linyi University, Linyi 276000, P. R. China; ∇ Nanotechnology Centre, Centre for Energy and Environmental Technologies (CEET), VŠB–Technical University of Ostra-va, 17. listopadu 2172/15, Ostrava-Poruba 708 00, Czech Republic; ○ Max Planck Institute for Polymer Research, Mainz 55128, Germany; ◆ Key Laboratory of Synthetic and Biological Colloids, Ministry of Education, School of Chemical and Material Engineering, 66374 Jiangnan University, Wuxi 214122, P. R. China

**Keywords:** covalent bridging strategy, triazine polymer, directed charge transfer, metal-free electrocatalyst, oxygen reduction reaction, Zn-air battery

## Abstract

Covalent triazine framework (CTF) derivatives have emerged as promising metal-free electrocatalysts due to their high nitrogen content and intrinsic porosity. However, their performance remains limited by sluggish interfacial charge transport and the inaccessibility of active sites. Herein, we report an interfacial covalent bridging strategy based on grafting polymerization to construct a carbon heterostructure electrocatalyst, featuring vertically aligned nitrogen-doped nanosheets covalently anchored onto graphene (v-N/CNS/Gr) support. The covalently bridged interface promotes interfacial charge transfer across the heterostructure, activating otherwise dormant nitrogen active sites and amplifying the oxygen reduction reaction (ORR) reactivity. *In situ* spectroscopic analyses and theoretical simulations reveal that the covalent bridged bonding promotes charge transport and oxygen activation, and optimizes the adsorption/desorption of intermediates, collectively contributing to reduced energy barriers along the 4e^–^ ORR pathway. As a result, the v-N/CNS/Gr delivers excellent ORR activity with a half-wave potential of 0.85 V (*vs* RHE). When employed as the cathode in a Zn-air battery, v-N/CNS/Gr achieves a high-power density and stable operation over 850 h. This work demonstrates a generalizable triazine-polymer-based interfacial bridge strategy for enhancing active site accessibility and charge transport in metal-free electrocatalysts.

## Graphical abstract

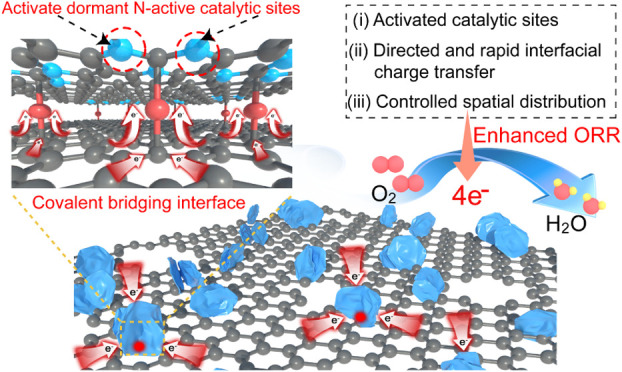

## Introduction

The accelerating proliferation of portable electronic devices highlights the urgent demand for advanced and operationally safe battery technologies.^1–3^ Among various contenders, zinc (Zn)-air batteries have emerged as an attractive technology owing to their abundant Zn resources, environmental benignity, and high energy density derived from the open-system architecture of air cathodes.^4–7^ Nevertheless, the sluggish oxygen reduction reaction (ORR) kinetics at the cathode are a major bottleneck hindering the practical application of Zn-air systems.^8–10^ To date, noble metal electrocatalysts have long been recognized for their exceptional intrinsic activity toward ORR. However, their high cost and susceptibility to fuel crossover have prompted extensive efforts to develop cost-effective alternatives with high activity and long-term durability.^11–13^ In response, massive progress has been made toward designing metal-free alternatives, among which, heteroatom-doped carbon nanocomposites have shown considerable promise due to their tunable surface chemistry and favorable electronic properties.^14–21^ Therefore, the development of highly active and cost-effective metal-free electrocatalysts is essential.

Covalent triazine frameworks (CTFs), an emerging class of highly cross-linked polymers containing triazine units and nanopores, have been considered as versatile platforms for constructing metal-free electrocatalysts. CTFs are characterized by high nitrogen content, tunable framework structures, and high surface area, which endow them with a unique ability to host catalytically active sites in a spatially controlled manner.^10,22–24^ Upon pyrolytic transformation, CTF-derived materials can retain nitrogen functionalities, particularly pyridinic and graphitic N, known to be catalytically favorable for ORR.^25,26^ Nonetheless, the practical deployment of CTF-based materials is often hindered by severe aggregation, which encapsulates nitrogen active sites and disrupts electron pathways, effectively reducing the availability of catalytically active sites. To address these issues, it is necessary to develop structural strategies that enhance charge transport and maximize the utilization of active sites. In this context, carbon-based heterostructures have been explored widely, with the aim of promoting spatial charge separation and transfer along the vertical direction. However, such conventional interfaces relying on weak van der Waals interaction suffer from severe aggregation, random stacking, and poor interfacial contact, which lead to inefficient charge transport and low utilization of nitrogen active sites (Figure 1a). Constructing covalent interfacial bridges within heterostructures offers an effective pathway to overcome these limitations. These architectures are expected to enhance interfacial interactions across heterogeneous domains, improve charge transfer and structural stability, and enhance active site exposure, thereby providing a promising structural foundation for high catalytic performance. Despite their potential, the rational design and controlled synthesis of CTF-derived carbon nanomaterials with covalently integrated heterointerfaces remain underexplored, and the structure–performance relationship governed by interfacial bridging in such system warrants further investigation.

**Figure 1:**
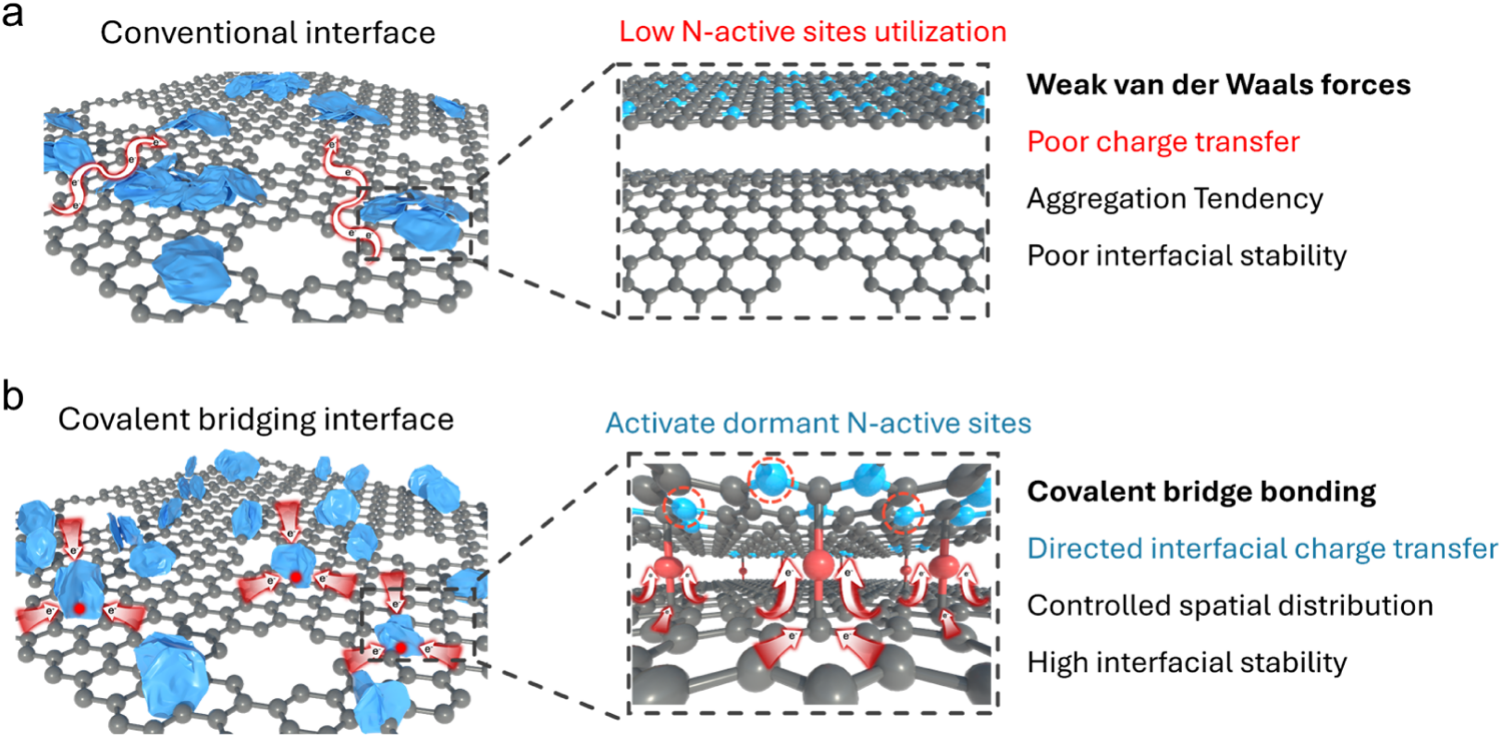
Schematic illustration of the interfacial covalent bridging strategy in heterostructure composites. (a) Conventional interfaces rely on weak van der Waals interactions, resulting in poor charge transfer and aggregation, which collectively reduce N-active site utilization and interfacial stability. (b) Covalent bridging interfaces, in contrast, form robust covalent bridge bonding that enables directed interfacial charge transfer and controls spatial distribution, which activates dormant N-active sites and achieves high interfacial stability.

Herein, we develop an interfacial grafting polymerization approach to construct a covalently bridged carbon heterostructure composite, where vertically aligned nitrogen-doped carbon nanosheets are covalently anchored on graphene (v-N/CNS/Gr) through robust covalent bonds. Specifically, molecular “rivet” pregrafted onto graphene oxide (GO) acts as a polymerization initiator, guiding the *in situ* growth and cross-linking of triazine-based frameworks onto the GO support. This bottom-up synthesis yields an integrated heterostructure with a covalent bonding heterointerface that ensures a strong coupling effect between the v-N/CNS and the graphene support. Compared with conventional carbon heterostructures, in which the interface is often dominated by weak van der Waals interactions, the covalently constructed interface establishes robust chemical linkages between the constituent domains. Such covalent coupling not only reduces interfacial resistance and accelerates directional charge transfer across the heterostructure but also effectively modulates the local electronic environment. As a result, previously inactive or “dormant” nitrogen sites are transformed into catalytically active centers, thereby markedly amplifying the ORR reactivity (Figure 1b). Combined *in situ* spectroscopic investigations and theoretical simulations demonstrate that the covalently bridged bonding promotes charge transport and oxygen activation, and optimizes the adsorption/desorption of intermediates, collectively contributing to reduced energy barriers along the 4e^–^ ORR pathway. As a result, the optimized v-N/CNS/Gr delivers an appreciable half-wave potential with a nearly 4e^–^ pathway, excellent methanol tolerance, and long-term stability. The assembled Zn-air battery using v-N/CNS/Gr catalyst offers the superior power density and an excellent long-term lifespan over 850 h at 5 mA cm^–2^. This work offers a viable strategy for constructing covalently bridged carbon architecture and provides new insights into the molecular-level design of high-performance metal-free electrocatalysts.

## Results and Discussion

As schematically illustrated in Figure 2a, we present an interfacial grafting polymerization approach for constructing covalently bridged carbon heterostructure composites from CTFs, resulting in vertically aligned nitrogen-doped carbon nanosheets covalently anchored on graphene. First, cyanuric chloride (CC) was grafted onto GO through nucleophilic substitution reactions between the chlorine atoms of CC and the hydroxyl or carboxyl groups on the GO surface, forming molecular “rivet” sites. These preanchored reactive sites served as covalent anchors to guide the subsequent *in situ* polymerization of CTF precursors. Meanwhile, the introduction of these molecular rivets enables the spatially confined coassembly of piperazine (PZ) and CC monomers, yielding a CTF-functionalized GO intermediate (CTF-GO embryo).^27^ Subsequent copolymerization between CC and PZ promoted the growth of extended CTF networks, forming vertically aligned CTF nanosheet arrays covalently anchored onto the GO support (v-CTF-GO) composites. The covalent anchoring effect not only ensures the stable immobilization of CTF nanosheets on the GO surface but also effectively prevents detachment and aggregation, which are common issues in physically mixed composites. Upon pyrolysis, the CTF and GO are converted into nitrogen-doped carbon nanosheets (N/CNS), yielding the final v-N/CNS/Gr composite. During this process, the covalent anchoring linkers are preserved, forming covalent bridges that maintain the vertically aligned architecture of the nanosheets. This structural integrity ensures that the nitrogen-rich carbon framework remains well-dispersed. To elucidate the influence of the graphene support and the covalent bridging interface, two comparative samples were also prepared. The first, N/CNS, was derived from pyrolyzed pristine CTF, while the second, N/CNS@Gr, was obtained by pyrolyzing a physically mixed CTF@GO composite lacking a covalent linkage. Moreover, enabled by the spatial confinement effect of CTF rivets, the density of carbon nanosheet arrays on graphene was systematically tuned by varying the monomer feed ratio (*i.e*., the mass ratio of CC to GO, see Experimental Section for details) in the v-CTF-GO precursor, thereby tailoring the array architecture for optimal exposure of active sites.

**Figure 2:**
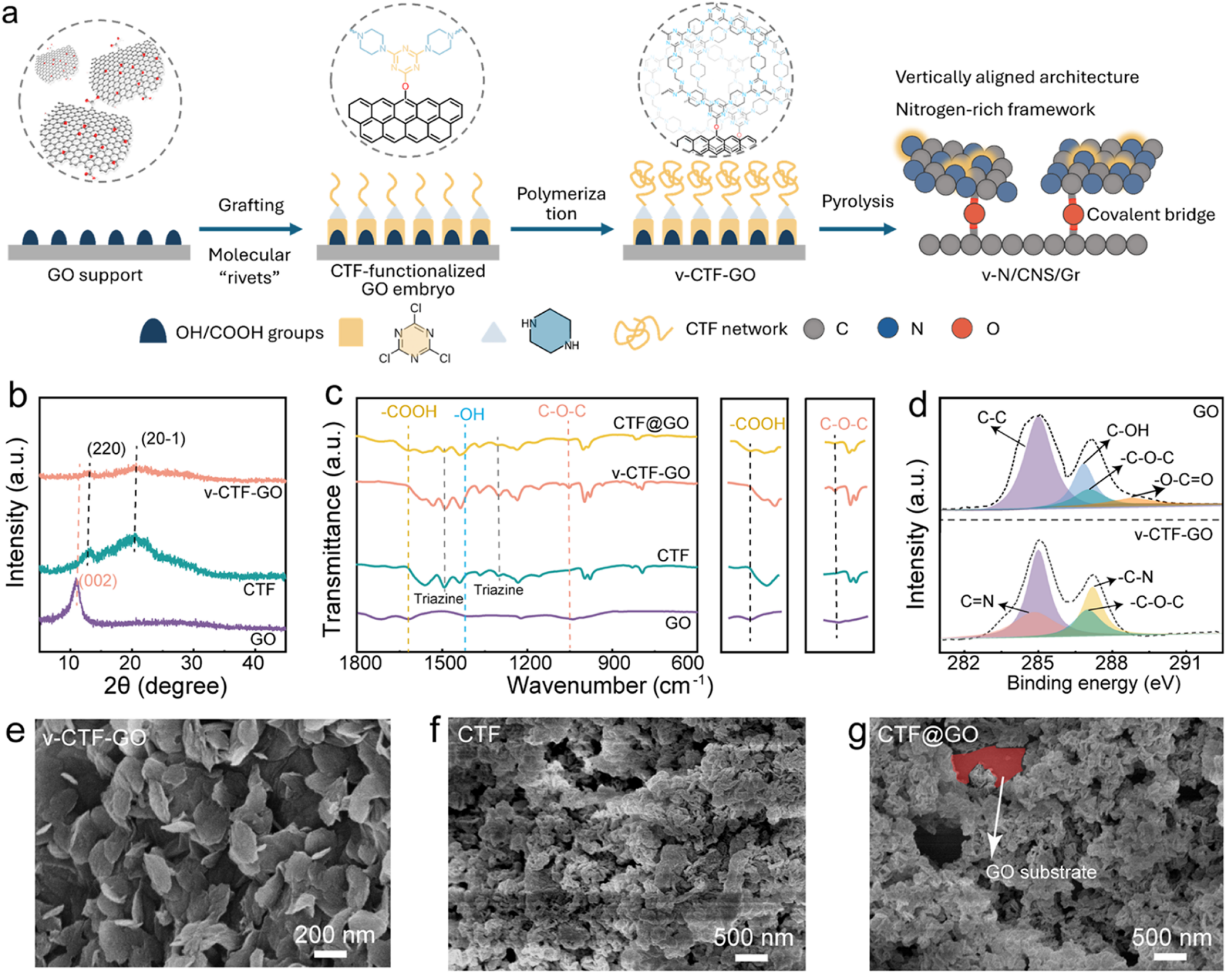
(a) Schematic illustration of the interfacial grafting polymerization approach for preparing v-N/CNS/Gr. (b) XRD patterns of GO, CTF, and v-CTF-GO. (c) FT-IR spectra of GO, CTF, CTF@GO, and v-CTF-GO. (d) XPS spectra of high-resolution C 1s for v-CTF-GO and GO. SEM images of (e) v-CTF-GO, (f) CTF, and (g) CTF@GO.

To confirm the formation of the v-CTF-GO composite and evaluate its structural features, a series of characterizations were performed. As shown in X-ray diffraction (XRD) patterns (Figure 2b), two characteristic peaks at about 13 and 21° can be indexed to the (220) and (20–1) planes of the CTF framework, indicating the formation of CTF on GO support.^28^ In the v-CTF-GO precursors (Figures 2 and S1), the characteristic peak at ∼11°, corresponding to the (002) plane of GO, is notably reduced. This attenuation suggests the establishment of covalent bonding between the CTF nanosheets and the GO support. Fourier transform infrared (FT-IR) spectra showed the disappearance of characteristic −OH and −COOH vibrations and the emergence of C–O–C and C–N stretching bands (Figure 2c), further validating the covalent linkage. This indicates that nucleophilic substitution occurred between the Cl atoms of CC and the OH/COOH groups on the GO support (Figure S2). In addition, a pronounced vibrational peak at 1050 cm^–1^ was observed in all v-CTF-GO precursors, corresponding to the characteristic C–O–C stretching vibration, further validating the formation of covalent linkages between the CTF network and the GO support. The peaks at 1490 and 1300 cm^–1^ are assigned to the stretching vibrations of C/N within the triazine rings.^29^ X-ray photoelectron spectroscopy (XPS) was conducted to determine the interfacial interaction between the CTF nanosheet and the GO support. The high-resolution C 1s XPS of v-CTF-GO in Figure 2d shows that the C–OH and COOH bonding in GO disappeared after grafting, indicating the covalent bonding formation between the CTF nanosheet and the GO support. The morphological structures of the different precursors were examined by scanning electron microscope (SEM). All v-CTF-GO samples reveal a nanosheet-array morphology on the GO support, with lateral dimensions ranging from 200 to 300 nm (Figures 2e and S3). For v-CTF-GO-1, prepared with 1 mmol of CC, the CTF nanosheets were dispersed sparsely on the GO surface (Figure S3a). Increasing the CC amount to 2 mmol resulted in the formation of vertically aligned nanosheet arrays on the GO support, forming v-CTF-GO. This observation suggests that a moderate amount of CC is beneficial for forming well-defined vertically aligned structures. However, when the CC dosage was further increased to prepare v-CTF-GO-3, excessive CTF growth resulted in densely packed and partially aggregated nanosheets on the GO surface (Figure S3b), which is detrimental to active site exposure and limits catalytic efficiency. For comparison, SEM images of pristine CTF and physically blended CTF@GO (Figure 2f,g) reveal only disordered, stacked nanosheet domains, highlighting the essential role of the covalent bridging interface in promoting spatial dispersion of CTF and enhancing the exposure of active sites. In addition, transmission electron microscopy (TEM) images of the v-CTF-GO with well-defined structures were acquired (Figure S4). The TEM images confirm that the CTF nanosheets are uniformly and tightly anchored on the GO surface.

Benefiting from the covalent bonding between the CTF and GO support, the nanosheet architecture of the v-CTF-GO precursor is well preserved during pyrolysis, resulting in vertically aligned N-doped carbon nanosheets covalently anchored onto graphene, as confirmed by SEM images in Figure S5. For v-N/CNS/Gr-1, no apparent nanosheet-array morphology is observed, indicating that low nanosheet densities are not effectively retained during pyrolysis (Figure S5a). By contrast, an optimal grafting density results in a stable, vertically aligned array structure for v-N/CNS/Gr (Figure S5b). Nevertheless, further increasing the nanosheet density brings nanosheets into severe aggregations during pyrolysis (Figure S5c), drastically reducing the specific surface area and accessible active sites. Notably, TEM images of v-N/CNS/Gr reveal the uniform growth of N/CNS on the graphene support, indicating retention of the vertically aligned architecture (Figure S6a). High-resolution TEM (HR-TEM) image further indicates a loosely packed network of ultrathin carbon nanosheets uniformly distributed on the graphene surface (Figure S6b). Such an ultrathin two-dimensional (2D) architecture is highly desirable to shorten the mass diffusion and charge transfer pathway during electrochemical reactions. The covalent bridged heterointerfaces of v-N/CNS/Gr can be further confirmed by the electron energy loss spectroscopy (EELS) of the K-edge of the O element at the specifically selected regions of scanning TEM (STEM) images (Figure 3a). The signals collected from v-N/CNS/Gr exhibit prominent C–O–C bands, whereas peaks corresponding to O species are negligible in N/CNS@Gr (Figure 3b). The comparison demonstrates the existence of the C–O–C heterointerface in v-N/CNS/Gr. The high-angle annular dark field (HAADF) energy-dispersive spectroscopy (EDS) elemental mappings are present in Figure 3c. The uniform distributions of C, N, and O are observed throughout v-N/CNS/Gr, confirming an efficient combination of v-N/CNS and the graphene support. The vertically aligned structure of v-N/CNS/Gr endows strong interfacial interactions between N/CNS and graphene with favorable distributions, in sharp contrast to the severe aggregation observed in N/CNS and N/CNS@Gr (Figure S7). Particularly, the vertically aligned v-N/CNS/Gr not only maximizes exposure of active sites but also facilitates the charge transfer due to interfacial couplings between v-N/CNS and graphene, thus contributing to electrocatalytic performance. We further conducted XRD to investigate the structural evolution after pyrolysis. As showed in Figures 3d and S8, all of the samples, including v-N/CNS/Gr, N/CNS@Gr, N/CNS, v-N/CNS/Gr-1, and v-N/CNS/Gr-3, reveal two broad diffraction peaks at 2θ ≈ 25 and 44°, attributed to the (002) diffraction plane of graphitic carbon and (010) diffraction plane of disordered carbon.^30^

**Figure 3:**
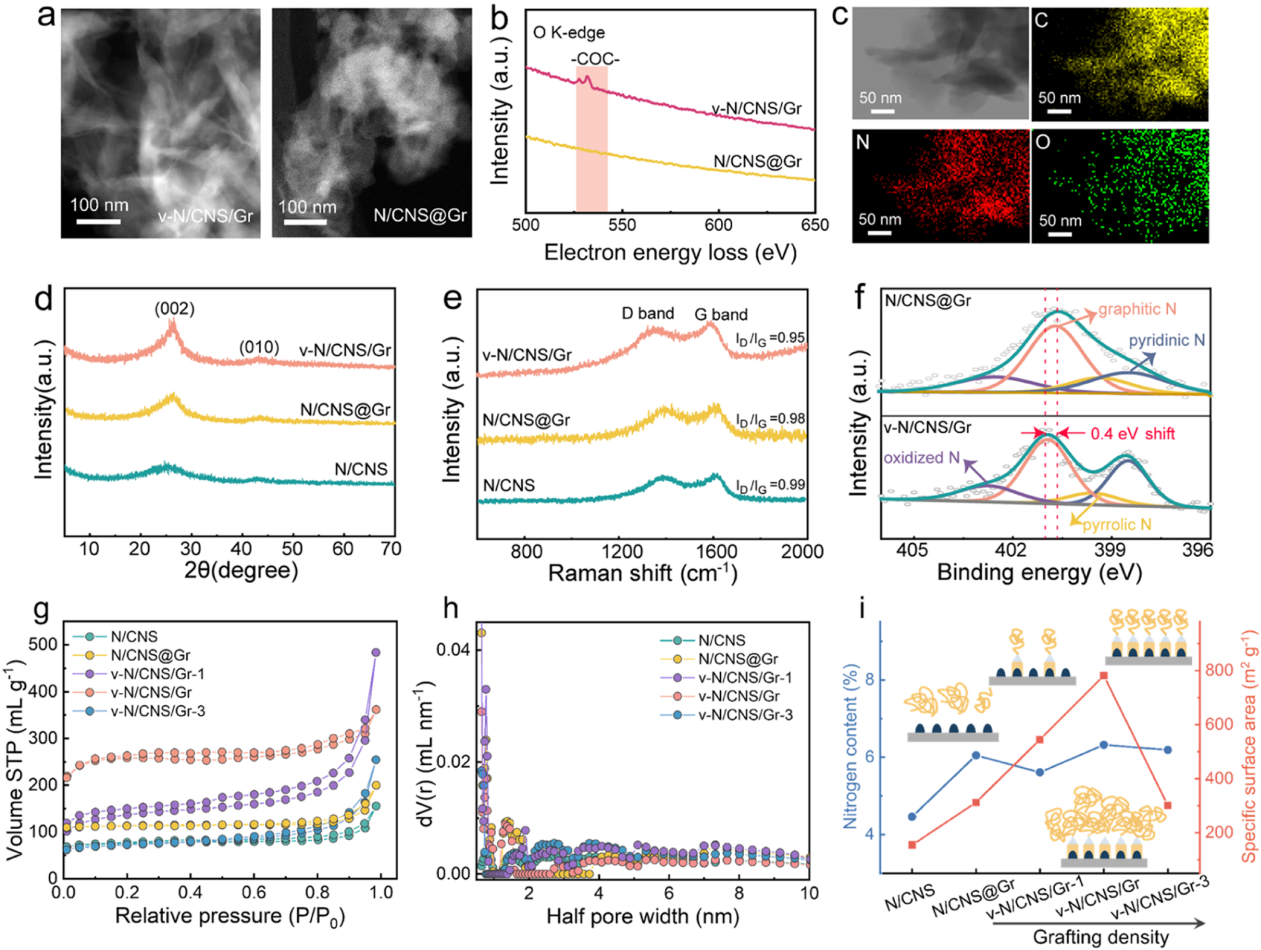
(a, b) STEM images and corresponding EELS spectra of O K-edge of v-N/CNS/Gr and N/CNS@Gr. (c) STEM image of v-N/CNS/Gr and corresponding HAADF-EDS mapping images of C, N, and O elements. (d) XRD patterns and (e) Raman spectra of N/CNS, N/CNS@Gr, and v-N/CNS/Gr. (f) High-resolution XPS N 1*s* spectra of N/CNS@Gr and v-N/CNS/Gr. (g) Nitrogen sorption isotherms and (h) the corresponding pore size distribution of N/CNS, N/CNS@Gr, and v-N/CNS/Gr with varying grafting density. (i) Relationship between nitrogen content (blue line) and specific surface area (red line) for N/CNS, N/CNS@Gr, and v-N/CNS/Gr with varying grafting density. The insets illustrate the prepyrolysis structures of CTF@GO (without a covalent bridged interface) and v-CTF-GO with different grafting densities, highlighting the transition from low-density, sparsely distributed structures (v-CTF-GO-1) to high-density, densely packed arrays (v-CTF-GO-3).

The degree of graphitization of the synthesized samples was evaluated using Raman spectroscopy, among which all samples showcase a distinct defective band (D) and graphitic band (G) located at 1350 and 1590 cm^–1^, respectively.^31,32^ The intensity ratio of the D band to the G band (*I*_D_/*I*_G_) is presented in Figures 3e and S9. Among them, v-N/CNS/Gr exhibits the lowest value of *I*_D_/*I*_G_, indicating its highest degree of graphitization. As shown in Figure S10, full-survey XPS spectra of all catalysts exhibit characteristic C 1s, N 1s, and O 1s signals, confirming the presence of nitrogen species associated with catalytically active sites. To identify the types of nitrogen functionalities contributing to catalytic activity, high-resolution N 1s spectra were deconvoluted into four peaks centered at 398.3, 399.5, 400.8, and 402.6 eV, corresponding to pyridinic N, pyrrolic N, graphitic N, and oxidized N, respectively.^33,34^ The elemental contents of the synthesized samples are summarized in Table S1. Notably, v-N/CNS/Gr exhibits a higher proportion of pyridinic and graphitic N species, which are recognized as active sites for the ORR (Figures 3f, S11, S12, and Table S2). Compared to N/CNS@Gr, the binding energy of v-N/CNS/Gr shifts positively by 0.4 eV, indicating that the covalently bridged heterointerfaces facilitate charge transfer. N_2_ adsorption–desorption isotherms were conducted to determine the specific surface area and pore size distribution of as-prepared samples. As shown in Figure 3g, all samples display typical type-IV isotherms, indicating the coexistence of micro- and mesopores, as further confirmed by the corresponding pore size distribution profiles (Figure 3h). The Brunauer–Emmet–Teller (BET) results of the as-prepared samples are summarized in Table S3. Among all of the samples, v-N/CNS/Gr presented the highest specific surface area (781.4 m^2^ g^–1^) and pore volume (0.897 mL g^–1^).

To further explore the influence of grafting density on the structural and chemical properties of covalent-bridged heterostructures, we systematically summarize the nitrogen content, specific surface area, and pore structure of the prepared samples, as shown in Figure 3i. The schematic insets illustrate the prepyrolysis precursor structure of CTF@GO (without covalent bridging) and v-CTF-GO with varying grafting densities. As the grafting density increases from v-N/CNS/Gr-1 to v-N/CNS/Gr-3, the specific surface area initially rises, reaching a peak at v-N/CNS/Gr, and then declines sharply at higher densities. This trend reflects the balance between nanosheet dispersion and aggregation. At moderate grafting densities of v-N/CNS/Gr, the formation of a vertically aligned, covalently bridged interface leads to a highly porous, interconnected network, potentially enhancing mass transport and active site accessibility. In contrast, excessive grafting leads to densely packed structures that limit surface accessibility. Similarly, the nitrogen content exhibits a comparable dependence on grafting density, peaking at v-N/CNS/Gr before declining as the nanosheet arrays become overcrowded. This indicates that moderate grafting densities optimize the porosity property and promote the formation of a higher density of active N species. It can be found that the covalently bridged interface in v-CTF-GO precursor stabilizes the vertically aligned architecture between the v-N/CNS and the Gr support and prevents nanosheet detachment and aggregation during pyrolysis, preserving the high specific surface area and active site exposure. In contrast, physically mixed CTF@GO results in disordered, stacked nanosheets with significantly lower accessible surface areas of N/CNS@Gr. These observations indicate that moderate grafting densities, combined with covalent bridging, are favorable for constructing highly porous, nitrogen-rich carbon heterostructures, providing a promising structural design for achieving high catalytic activity.

To elucidate the role of interfacial covalent bridging, the electrocatalytic performance of v-N/CNS/Gr (with covalent bridge) was evaluated in O_2_-saturated 0.1 M KOH using a standard three-electrode system, v-N/CNS/Gr-1, v-N/CNS/Gr-3, N/CNS@Gr (without covalent bridge), N/CNS, and commercial Pt/C tested under identical conditions for comparison. Cyclic voltammogram (CV) measurements were performed in electrolytes saturated with O_2_ and N_2_, respectively. Figure S13 reveals that v-N/CNS/Gr shows an obvious reduction peak in the O_2_-saturated media, whereas no reduction peak is observed in the N_2_-saturated media, demonstrating a significant ORR catalytic activity for the as-prepared electrocatalysts.^35^ Notably, the v-N/CNS/Gr exhibits a more positive reduction peak compared with its counterparts, indicating superior ORR catalytic performance (Figure 4a). Linear sweep voltammetry (LSV) polarization measurements were conducted for all as-prepared samples. Among them, v-N/CNS/Gr demonstrated an enhanced ORR performance with a half-wave potential of 0.85 V (*vs* RHE) and limiting current density (*J*_L_) of –5.7 mA cm^–2^, outperforming its counterparts and approaching the performance of commercial Pt/C (Figure 4b). These observations suggest that the presence of a covalent bridging interface in v-N/CNS/Gr contributes to the improved ORR kinetics.

**Figure 4:**
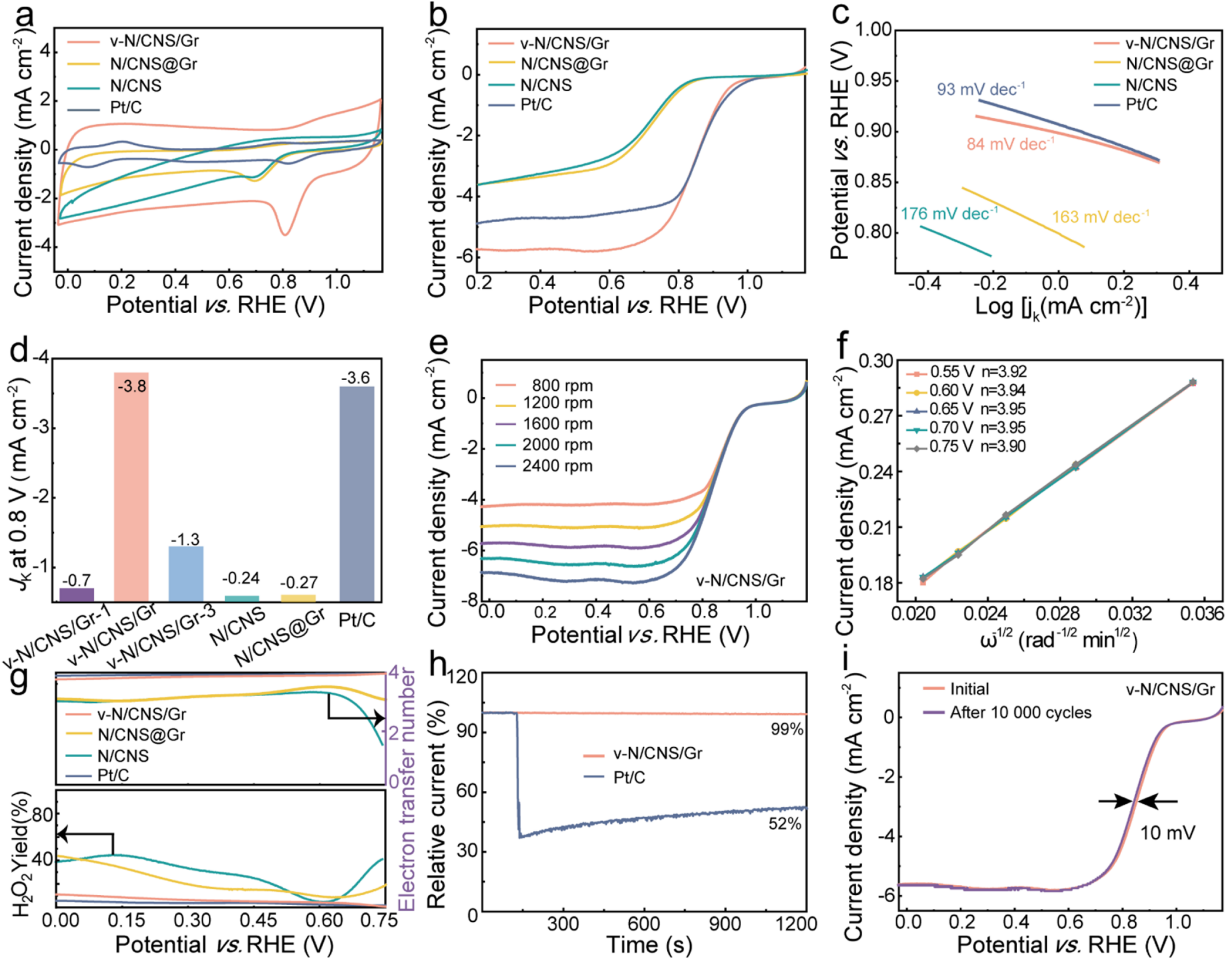
(a) CV curves, (b) LSV curves, and (c) Tafel plots of v-N/CNS/Gr, N/CNS@Gr, N/CNS, and Pt/C. (d) *J*_k_ at 0.8 V (*vs* RHE) of v-N/CNS/Gr with grafting density, N/CNS@Gr, N/CNS, and Pt/C. (e) LSV curves at different rotating speeds, and (f) corresponding K–L plots at various potentials of v-N/CNS/Gr. (g) H_2_O_2_ yields rate and electron transfer number of v-N/CNS/Gr, N/CNS@Gr, N/CNS, and Pt/C. (h) Normalized *i*–*t* curves of v-N/CNS/Gr and Pt/C with an additional 3 M methanol. (i) The ORR catalytic activity of v-N/CNS/Gr after 10,000 CV cycles.

To further evaluate the ORR kinetics, Tafel slopes were obtained from the corresponding polarization profiles. As shown in Figures 4c, S14 and S15, v-N/CNS/Gr displays a Tafel slope of 84 mV dec^–1^, markedly lower than v-N/CNS/Gr-1 (136 mV dec^–1^), v-N/CNS/Gr-3 (152 mV dec^–1^), N/CNS@Gr (163 mV dec^–1^), N/CNS (176 mV dec^–1^), and Pt/C (93 mV dec^–1^), respectively.^36^ Notably, the ORR performance displays a volcano-type trend concerning grafting density, with v-N/CNS/Gr achieving an optimal balance that maximizes active site accessibility and facilitates charge transport. These results indicate that the distribution and density of grafted CTF precursors play a crucial role in regulating catalytic site exposure, thereby significantly influencing the overall ORR activity. Electrochemical impedance spectroscopy (EIS) further confirms that v-N/CNS/Gr exhibits the lowest charge-transfer resistance (*R*_ct_) among all tested samples (Figure S16), consistent with its highest electrical conductivity (Table S4), indicating that constructing covalently bridged carbon heterostructures is beneficial for charge transfer. Meanwhile, v-N/CNS/Gr delivers the highest kinetic current density (*J*_k_) at 0.8 V (*vs* RHE), further suggesting its enhanced ORR kinetics, which can be attributed to its well-defined nanoarchitecture and maximized active site exposure (Figure 4d). We compared the ORR catalytic performance of v-N/CNS/Gr with that of other recently reported metal-free ORR catalysts (Table S5), and confirmed its outstanding activity.^37–50^ Moreover, the LSV profiles were carried out at different rotation speeds using a rotating disk electrode (RDE) to further probe the ORR kinetics. Figure 4e displays that *J*_L_ increases with rotation rate, indicating that the ORR kinetics is dominated by the diffusion of the O_2_ diffusion. Additionally, Koutecky–Levich (K–L) plots were employed to assess the electron transfer number (*n*) for v-N/CNS/Gr. The average n is calculated to be 3.93 (Figure 4f), evidencing a nearly four-electron transfer pathway for the ORR process. This is further corroborated by rotating ring-disk electrode (RRDE) measurements, which reveal a low H_2_O_2_ selectivity of approximately 5% (Figures 4g and S17). Impressively, v-N/CNS/Gr demonstrates excellent methanol tolerance, exhibiting only a slight decline in the current density upon methanol exposure, in contrast to the significant current drop observed for the Pt/C benchmark (Figure 4h). Likewise, the durability of v-N/CNS/Gr was conducted via a rapid accelerated durability measurement involving CV testing. As illustrated in Figure 4i, after 10,000 CV cycles, the half-wave potential of v-N/CNS/Gr shifts by only 10 mV negatively, underscoring its exceptional long-term stability. Additional durability assessment was performed via a relative retention *i*–*t* testing, and the v-N/CNS/Gr emerges with an excellent stability with a current retention of 98% after 45,000 s of continuous operation, compared to only 86% for the Pt/C benchmark (Figure S18). The enhanced ORR performance of v-N/CNS/Gr can be due to the covalently bridged carbon heterostructure, which features a high specific surface area, superior electrical conductivity, and abundant active sites. Therefore, the interfacial bridging between v-N/CNS and graphene support facilitates directed charge transfer, and promotes extensive active site exposure. In addition, to clarify the stability of interfacial covalent bridges in v-N/CNS/Gr, we analyzed the composition and morphology of the v-N/CNS/Gr after stability testing. As shown in Figure S19, the TEM image, XRD pattern, and XPS spectrum of v-N/CNS/Gr are analogous to those before testing, indicating that the interfacial covalent bridges in v-N/CNS/Gr achieve excellent stability.

To uncover the fundamental origin of enhanced ORR activity induced by interfacial covalent bridging in v-N/CNS/Gr, density functional theory (DFT) calculations were conducted on three representative structural models, including v-N/CNS/Gr, N/CNS@Gr, and N/CNS (Figure S20). As exhibited by the projected densities of states (PDOS) in Figure 5a, the N 2p p-band center in v-N/CNS/Gr shifts to a moderate energy relative to the Fermi level, compared to N/CNS@Gr and N/CNS. This optimized electronic configuration balances adsorption and desorption of reaction intermediates, enhancing surface reactivity and accelerating ORR kinetics. Figure 5b presents the proposed 4e^–^ ORR reaction pathway for v-N/CNS/Gr, while the configurations of ORR intermediates for N/CNS@Gr and N/CNS are displayed in Figures S21 and S22. The proposed 4e^–^ ORR mechanism mainly involves four steps: the formation of *OOH, *O, and *OH intermediates, followed by the reduction to H_2_O.

**Figure 5:**
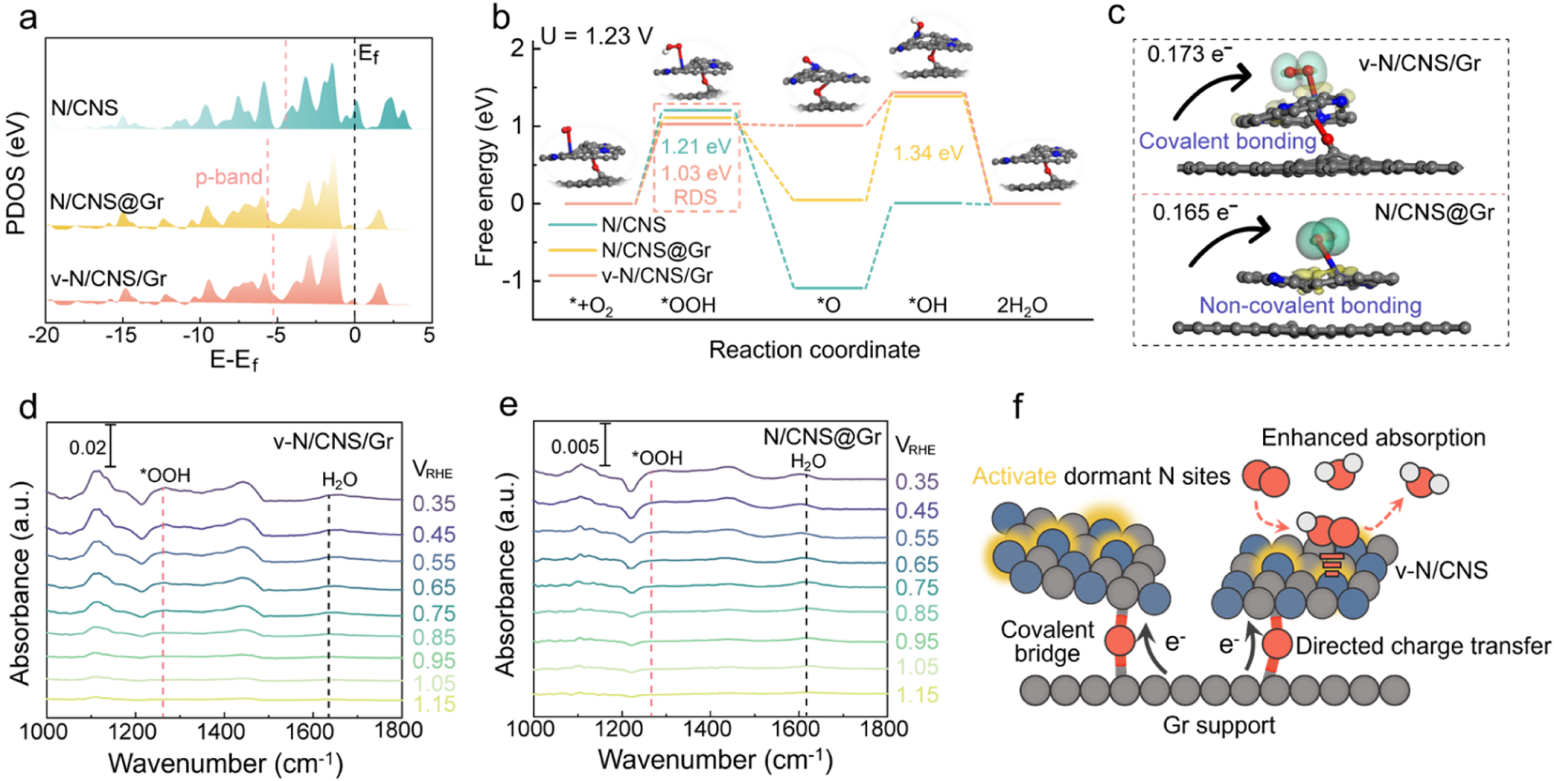
(a) PDOS of N 2p orbitals of N/CNS, N/CNS@Gr, and v-N/CNS/Gr. The black and red dashed lines indicate the Fermi level and p-band center of the N 2p orbitals, respectively. (b) Free energy diagrams of the ORR pathway on N/CNS, N/CNS@Gr, and v-N/CNS/Gr. (Inset: optimized structure of N/CNS@Gr). (c) Electron density difference maps of *O_2_ adsorbed on v-N/CNS/Gr (top) and N/CNS@Gr (bottom). The yellow and blue regions are regions with increased and decreased electron density, respectively. The C, N, O, and H elements are colored in gray, blue, red, and white, respectively. *In situ* ATR-SEIRAS spectra of (d) v-N/CNS/Gr and (e) N/CNS@Gr during the ORR process. (f) Schematic illustration of the covalently bridged v-N/CNS/Gr. Covalent bridge activates dormant N sites, facilitates directed charge transfer, and promotes efficient *OOH adsorption, collectively contributing to improved ORR activity.

Free energy diagrams were calculated at 1.23 V for v-N/CNS/Gr, N/CNS@Gr, and N/CNS, as presented in Figure 5b. The rate-determining step (RDS) for v-N/CNS/Gr is the formation of the *OOH intermediate. Among them, the v-N/CNS/Gr model demonstrates the lowest formation energy of *OOH at 1.03 eV, compared to 1.11 eV for N/CNS@Gr and 1.21 eV for N/CNS, respectively. In addition, the formation energy of *OH on v-N/CNS/Gr significantly reduced (0.43 eV) relative to N/CNS@Gr (1.34 eV) and N/CNS (1.10 eV), respectively. These results underscore that the covalently bridged heterostructure of v-N/CNS/Gr effectively lowers the energy barrier of the RDS and promotes the formation of *OH, thereby further facilitating reactions toward the 4e^–^ ORR process. To further identify the actual active sites in v-N/CNS/Gr, free energy diagrams were calculated for reactions occurring at oxygen sites. As shown in Figure S23, the RDS at the oxygen sites is the desorption of H_2_O with an energy barrier of 3.20 eV, which is much higher than that at the nitrogen sites with RDS of the formation of the *OOH intermediate with 1.03 eV. This substantial difference indicates that the ORR process in v-N/CNS/Gr preferentially occurs at nitrogen sites rather than oxygen sites. Moreover, to further clarify the role of interfacial covalent bonding, Mulliken charge analysis was conducted to quantify the charge redistribution within the heterostructures (Figure S24). Figure 5c presents the electron density difference maps for v-N/CNS/Gr and N/CNS@Gr with *O_2_ adsorbed. For v-N/CNS/Gr, a higher charge transfer of 0.173 e^–^ to O_2_ was observed, in contrast to 0.165 e^–^ for N/CNS@Gr, indicating more efficient electron donation. This enhanced electron donation highlights the favorable role of covalent interfacial bonding among carbon heterostructures in promoting charge transfer during the ORR process.^51^ Additional electron density difference maps for N/CNS, N/CNS@Gr, and v-N/CNS/Gr with *OOH and *OH adsorbed are shown in Figures S25 and S26, indicating a beneficial effect of covalent bridge bonds among carbon heterostructures.

To investigate the effect of the covalently bridged interface on interfacial mass transport and intermediate evolution, we conducted *in situ* attenuated total reflection surface-enhanced infrared absorption spectroscopy (ATR-SEIRAS) on v-N/CNS/Gr and N/CNS@Gr (Figure 5d,e). A characteristic vibrational peak at 1272 cm^–1^, corresponding to the adsorbed *OOH intermediate on v-N/CNS/Gr, emerges across a wide potential window of 0.95–0.35 V during the ORR process. In contrast, for N/CNS@Gr, the *OOH peak appears over a narrower potential range of 0.75–0.35 V and with lower intensity, suggesting that v-N/CNS/Gr manifests stronger *OOH adsorption compared to the N/CNS@Gr. This observation is consistent with the PDOS results in Figure 5a, further supporting the enhanced intermediate stabilization on v-N/CNS/Gr. The attenuated *OOH adsorption on N/CNS@Gr renders the formation of the *OOH intermediate thermodynamically less favorable, impeding the following reduction reaction steps and suppressing overall ORR catalytic activity.^52^ The *in situ* ATR-SEIRAS results demonstrate that the boosted ORR catalytic activity originates from the stronger adsorption of *OOH on v-N/CNS/Gr. These findings demonstrate that the covalent bridged interface in v-N/CNS/Gr establishes an efficient electronic coupling channel through robust bridging bonds, facilitating directional electron transfer from the graphene support to the N/CNS. This interfacial charge redistribution not only enhances overall conductivity but also activates otherwise dormant nitrogen active sites by precisely tuning their local electronic environments, thereby promoting efficient *OOH adsorption during the ORR process (Figure 5f).

Inspired by the excellent ORR activity of the v-N/CNS/Gr, oxygen evolution reaction (OER) measurements were further carried out in an O_2_-saturated KOH solution. As depicted by the LSV profiles (Figure S27), the v-N/CNS/Gr exhibited an overpotential of 0.25 V at a current density of 10 mA cm^–2^, comparable to that of commercial IrO_2_. Encouraged by the superior ORR and OER performance, a self-assembled Zn-air battery employing v-N/CNS/Gr was fabricated to demonstrate its practical application potential. A schematic illustration of the Zn-air battery configuration is shown in Figure 6a. The Zn-air battery using v-N/CNS/Gr affords a high and stable open-circuit voltage of 1.53 V, surpassing that of commercial Pt/C + IrO_2_ of 1.41 V (Figure 6b). Besides, three liquid Zn-air batteries assembled using v-N/CNS/Gr were connected in series and successfully powered a 4 V light-emitting diode (inset of Figure 6b), indicating its excellent discharge capability. Notably, the galvanostatic discharge profiles at 10 mA cm^–2^ deliver a specific dis-capacity of 793 mAh g_Zn_^–1^, exceeding that of the commercial Pt/C with 730 mAh g_Zn_^–1^ (Figure S28). As shown in Figure 6c, the Zn-air battery with v-N/CNS/Gr demonstrates improved rate capability, maintaining stable voltage plateaus across a broad range of current densities of 10, 20, 30, 40, and 50 mA cm^–2^ and fully recovers to the initial state when cycled back to 10 mA cm^–2^. Most impressively, the discharge polarization and power density profiles of the Zn-air battery using v-N/CNS/Gr reveal a peak power density of 265.3 mW cm^–2^, which is much better than that of N/CNS@Gr (77 mW cm^–2^, Figure S29), and even nearly three times that of the Pt/C + IrO_2_ counterpart (93.6 mW cm^–2^), representing one of the highest values reported to date for metal-free air cathodes in Zn-air batteries (Figure 6d). In addition, long-term durability tests at 5 mA cm^–2^ (Figure 6e) reveal that the v-N/CNS/Gr catalyst supports superior operational stability for over 850 h with negligible performance decay with a stable voltage profile. In stark contrast, the Pt/C + IrO_2_ cell exhibits premature failure after ∼190 h with large voltage spikes. In the realm of research for aqueous Zn-air batteries, this study stands out for its power density and durability, outperforming most reported Zn-based hybrid systems (Table S6). These results not only highlight the robustness of the covalently bridged interfacial architecture but also position v-N/CNS/Gr as a compelling alternative to noble metal-based catalysts for high-performance, sustainable energy conversion and storage applications.

**Figure 6:**
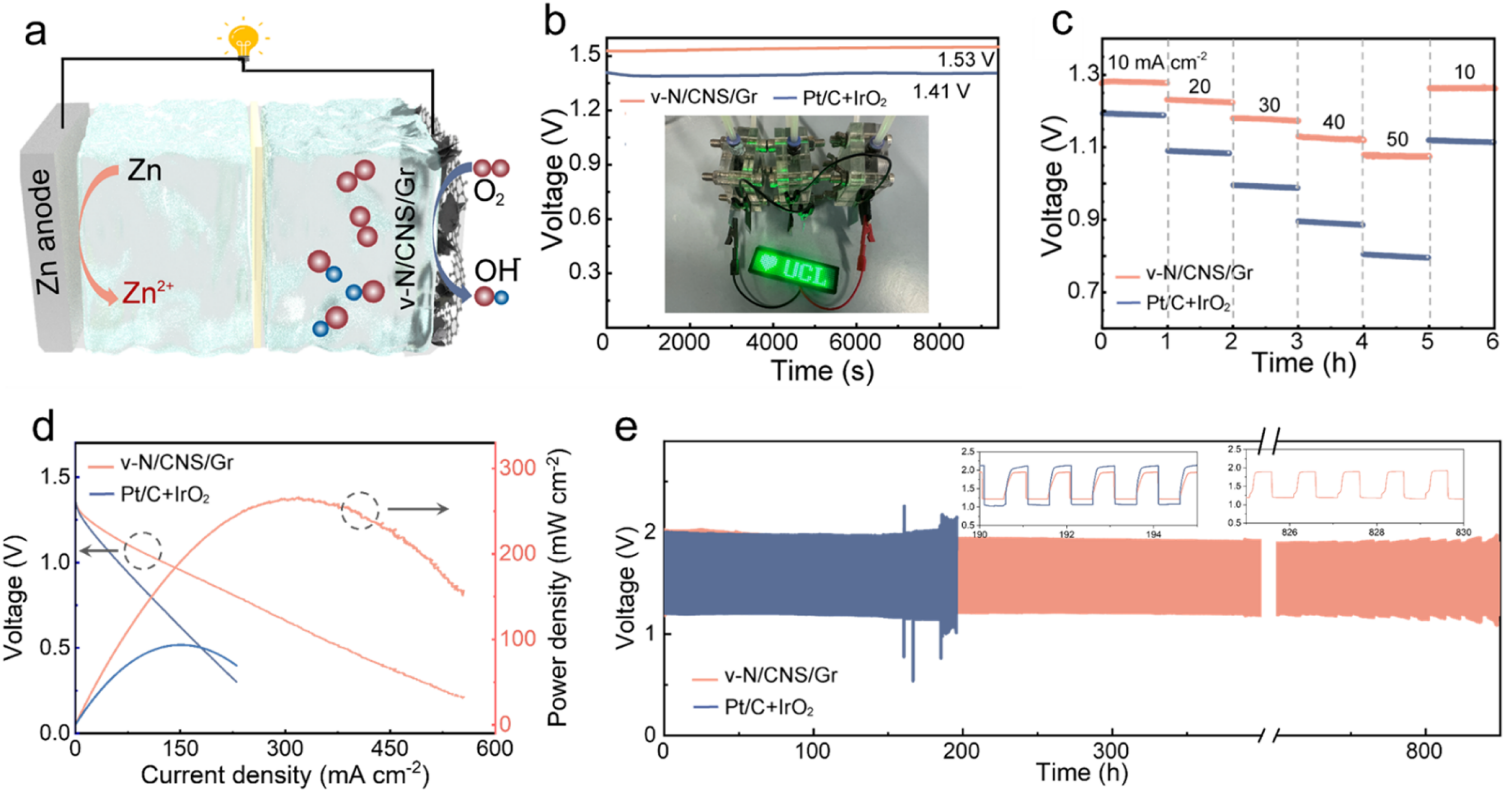
(a) Schematic illustration of the assembled Zn-air battery configuration. (b) Open-circuit voltage measurements of Zn-air batteries using v-N/CNS/Gr and Pt/C + IrO_2_ electrodes. Inset: photography of a green light-emitting diode powered by a three-series-connected Zn-air battery module using v-N/CNS/Gr. (c) Rate-performance, (d) discharge polarization curves and corresponding power density, and (e) stability performance of the Zn-air batteries using v-N/CNS/Gr and Pt/C + IrO_2_ electrodes. Inset of panel (e) shows the enlarged profiles.

## Conclusions

In summary, we developed an interfacial grafting polymerization strategy to construct a covalently bonded heterointerface between vertically aligned nitrogen-doped carbon nanosheets and a graphene support, enabling the formation of a well-integrated v-N/CNS/Gr nanocomposite upon pyrolysis. This covalently bonded heterointerface ensures directed and efficient interfacial charge transfer pathways and maximizes utilization of catalytic sites, confirmed by the calculations and *in situ* ATR-SEIRAS results. As a result, the optimized v-N/CNS/Gr electrocatalyst demonstrates an outstanding electrochemically catalytic activity toward the ORR, achieving an enhanced ORR activity of an improved half-wave potential and long-term operation stability. As a practical demonstration, the assembled Zn-air battery device employing v-N/CNS/Gr as the air cathode exhibits a high-power density of 265.3 mW cm^–2^ and an excellent long-term lifespan over 850 h. This work opens valuable insight into the rational construction of metal-free electrocatalysts with highly catalytic activity and durability through covalently bridged carbon heterostructure modulation for advanced metal-air batteries. This work proposes a universal interfacial grafting strategy for constructing covalently bridged heterostructures that activate otherwise inert sites and optimize charge-transfer pathways, offering broad applicability to catalytic systems such as the oxygen evolution reaction, the hydrogen evolution reaction, and the carbon dioxide reduction reaction, etc.

## Experimental Section

### Synthesis of v-CTF-GO

Typically, 0.1 g of graphene oxide (GO) powder was synthesized by a modified Hummers method,^53^ and then dispersed in 10 mL of tetrahydrofuran (THF), followed by 30 min of ultrasonication to obtain a homogeneous dispersion. Meanwhile, A solution of 2 mmol of CC in 20 mL of THF was added to the GO dispersion and transferred to a 100 mL reflux flask under continuous stirring. Afterward, the above mixed solution was stirred at 0 °C for 30 min. Then, 3 mmol of PZ was added to the mixture. Subsequently, 8 mL of triethylamine (TEA) was added, and the reaction was performed sequentially at ∼0 °C (ice–water bath) for 2 h, at room temperature (∼25 °C) for 4 h, and at ∼97 °C (reflux) overnight, respectively. The precipitate was collected by filtration, washed several times with excess THF, ethanol, and deionized water, and then dried in a vacuum oven at 80 °C overnight, denoted as v-CTF-GO. Meanwhile, v-CTF-GO-1 and v-CTF-GO-3 were prepared by similar synthetic procedures using different monomer ratios (1 mmol of CC and 1.5 mmol of PZ for v-CTF-GO-1; 4 mmol of CC and 6 mmol of PZ for v-CTF-GO-3). For comparison, pristine CTF and a physically mixed CTF@GO composite (without a covalent linkage) were also prepared as control precursors.

### Synthesis of v-N/CNS/Gr

The as-synthesized v-CTF-GO were placed into a covered crucible and annealed at 1000 °C for 2 h under N_2_ atmosphere with a heating rate of 2 °C min^–1^ to obtain the electrocatalyst. After cooling to room temperature, the resulting powder was washed with deionized water, vacuum-dried overnight at 100 °C, and denoted as v-N/CNS/Gr. For comparison, nitrogen-doped carbon nanosheets (N/CNS) and N/CNS@Gr were prepared under the same calcination conditions using CTF and CTF@GO as pyrolytic precursors, respectively. Furthermore, the density of carbon nanosheet arrays on graphene was systematically tuned by pyrolyzing v-CTF-GO-1 and v-CTF-GO-3 precursors, yielding v-N/CNS/Gr-1 and v-N/CNS/Gr-3, respectively.

### Characterization

The morphology of the powder products was examined by field-emission scanning electron microscopy (FE-SEM, JEOL JSM-7001F). Transmission electron microscopy (TEM) and high-resolution TEM (HR-TEM) images were obtained on a JEOL JEM-2100. Energy-dispersive X-ray spectroscopy (EDS) elemental mappings were collected on a Talos F200X G2 TEM system operated at 120 kV. The Fourier transform infrared (FT-IR) spectra were measured with a Nicolet Impact 410 Fourier transform infrared spectrometer. The nitrogen adsorption/desorption isotherms were collected at the temperature of liquid nitrogen (77 K) utilizing a QUADRASORB SI automated surface area and pore size analyzer (Quantachrome Corporation). The specific surface area was calculated from the adsorption data according to the Brunauer–Emmett–Teller (BET) method. The pore size distribution was derived with nonlocal density functional theory (DFT) on the desorption branch. Prior to measurement, the samples were degassed under a vacuum at 150 °C for 24 h. X-ray diffraction (XRD) patterns were recorded on a Bruker D8 Advance diffractometer equipped with a Cu Kα radiation source. Raman spectra were recorded on a Renishaw inVia Raman spectrometer with a 532 nm laser. Ultraviolet visible (UV–vis) absorption spectra were measured on a Lambda 35 (PerkinElmer) spectrophotometer. X-ray photoelectron spectroscopy (XPS) measurements were carried out on a Kratos Axis ULTRA spectrometer. Elemental analysis was achieved with a Vario EL III Element analyzer based on a combustion method.

## Supplementary Material



## Notes

Detailed experimental procedures; characterizations; computational methods (PDF)
