# Supplementary material for: Covalent-Bridged Heterointerfaces via Grafted Triazine Organic Polymers Enable Directed Charge Transfer for Efficient Oxygen Reduction in Zn–Air Batteries
Source: ACS Nano. 2025 Aug 25;19(35):31870–81. doi: 10.1021/acsnano.5c11348 (PMC12424297; doi:10.1021/acsnano.5c11348)
Supplement: Supplementary file 1 [file nn5c11348_si_001.pdf]

Supporting Information

Covalent Bridged Heterostructures via Grafted Triazine Organic Polymers Enable Directed Charge Transfer for Efficient Oxygen Reduction in Zn–Air Batteries

Shan Chen,[†,‡] Jitao Shang,[§] Fei-er Peng,[†] Zihan Song,[∥] Yong Zheng,[,⊥] Yuhang Dai,[∥] Jiexin Zhu,[‡] Fei Guo,[‡] Xinliang Fu,[†] Kaibin Chu,[#] Xueying Cao,[*,#] Yue Ouyang,[*,‡] Ivan P. Parkin,[‡] Yazhou Zhou,[*,∇,○] Guanjie He,[‡] Tianxi Liu,[◆] and Wei Zong[*,‡,∥]*

[†]Hubei Key Laboratory of Pollutant Analysis & Reuse Technology, College of Chemistry and Chemical Engineering, Hubei Normal University, Huangshi 435002, P. R. China.

[‡]Christopher Ingold Laboratory, Department of Chemistry, University College London, 20 Gordon Street, London WC1H 0AJ, UK.

[§]Institute of Technological Sciences, Wuhan University, Wuhan, Hubei, 430072 China.

[∥]Department of Engineering Science, University of Oxford, Parks Road, Oxford OX1 3PJ, UK.

[⊥]College of Materials and Chemical Engineering, Key Laboratory of Inorganic Nonmetallic Crystalline and Energy Conversion Materials, China Three Gorges University, Yichang 443002, P. R. China.

[#]College of Materials Science and Engineering, Linyi University, Linyi 276000, P. R. China.

[∇]Nanotechnology Centre, Centre for Energy and Environmental Technologies (CEET), VŠB–Technical University of Ostra-va, 17. listopadu 2172/15, Ostrava-Poruba 708 00, Czech Republic.

[○]Max Planck Institute for Polymer Research, Mainz 55128, Germany.

[◆]Key Laboratory of Synthetic and Biological Colloids, Ministry of Education, School of Chemical and Material Engineering, Jiangnan University, Wuxi 214122, P. R. China.

Corresponding author: email: zhengyong@ctgu.edu.cn; caoxueying@lyu.edu.cn; yue.ouyang@ucl.ac.uk; yazhou@mpip-mainz.mpg.de; wei.zong@eng.ox.ac.uk

Experimental section

Chemical reagents and materials: Graphite powder (325 meshes) was attained from Alfa-Aesar. Triethylamine (TEA, ≥ 99.5%), tetrahydrofuran (THF, AR), cyanuric chloride (CC, 99%), piperazine (PZ, 99.5%), and potassium hydroxide (KOH ≥ 90%) were purchased from Aladdin. Sodium nitrate ($NaNO_3$, AR, ≥ 99.0%), sulfuric acid (H_2SO_4, 98%), potassium permanganate ($KMnO_4$, AR), hydrochloric acid (HCl, 37%), ethanol (99.5%) and hydrogen peroxide (H_2O_2, 30%) were procured from Sinopharm Chemicals. IrO_2 and 20 wt% commercial Pt/C were purchased from Alfa Aesar chemical Co, Ltd, China. Deionized (DI) water was used throughout the experiments. All the chemicals were used as received without any further purification unless specified.

Density functional theory (DFT) calculations: DFT calculations were employed by DMol3 in Material Studio with the plane-wave pseudopotential method for the first-principles-based quantum mechanics simulations.[2, 3] The exchange and correlation potentials were present in the generalized gradient approximation with the Perdewe-Burkee-Ernzerh of (GGA-PBE). To explore the reaction pathways of oxygen reduction reaction (ORR) and electronic properties, a defective graphene supercell consisting of 127 atoms was built. For N/CNS@Gr, a small nitrogen-doped carbon (N/CNS) sheet with 31 atoms is placed above the graphene sheet while there is a -O- bond between these two carbon layers for v-N/CNS/Gr. A vacuum slab of 15 Å was employed in the (001) direction to avoid the interaction influence of the periodic boundary conditions. Van der Waals (VDW) interactions were corrected using the DFT-D method of TS 22. A Γ-centred Monkhorst-Pack mesh with 1 × 1 × 1 K-points was used for Brillouin zone integration. During computation, the double numerical plus polarization functions (DNP = 4.4) were used as a basis set. The Kohn-Sham self-consistent field calculations were performed with convergence tolerance of 1×10^{-6} Hatree on the total energy.

For the Gibbs free energies (ΔG) calculations, the zero-point energy (ΔZPE) and entropy corrections are considered in the absorption energies parts.

$$\Delta G = \Delta E + \Delta ZPE - T\Delta S \quad (1)$$

in which ΔE is the difference of electronic energy in the ground-state obtained from self-consistent calculation. T is the temperature (T = 298.15 K). ΔS is the entropy difference. The entropies of molecules in the gas phase were taken from the NIST database.

Electrochemical measurements: The electrochemical measurements of the as-prepared catalysts were carried out on a CHI 660D electrochemical workstation (Shanghai, Chenhua Instruments) with a three-electrode configuration. The ORR catalytic activity of the electrocatalysts were tested via cyclic voltammetry (CV) with a scan rate of 50 mV s^{-1} and linear sweep voltammetry (LSV) with a scan rate of 10 mV s^{-1} on a rotating disk electrode (RDE, Pine AFMSRCE 2762). A glassy carbon disk was applied as the working, while a platinum (Pt) sheet and a saturated calomel electrode (SCE) were employed as counter electrode and reference electrodes, respectively. All potentials are converted to the reference hydrogen electrode (RHE) by the following formulas.

$$E\ (V\ \textit{vs.}\ RHE) = E\ (V\ \textit{vs.}\ SCE) + 0.244 + 0.0591*pH \quad (2)$$

The catalyst ink was prepared by dispersing 5 mg of the electrocatalyst into a mixed solution including 95 μL of 5 wt% Nafion solution and 350 μL of ethanol. Subsequently, 5 μL of the electrocatalyst ink was dropwise onto a grassy carbon electrode with electrocatalyst loaded of 0.285 mg cm^{-2}. The commercial Pt/C catalyst with the same loading mass was also investigated as benchmark for comparison. The polarization profiles were carried out in a 0.1 M O_2-saturated KOH electrolyte under a rotational speed of 1600 rpm at a potential range from 0.2 to 1.2 V (*vs.* RHE). The long-term stability and methanol tolerance test were both measured via using the chronoamperometric response in a 0.1 M O_2-saturated KOH. The electron transfer number (n) was calculated through the Koutecky-Levich (K-L) linear fitting equations using the following formulas:[4]

$$\frac{1}{J} = \frac{1}{J_L} + \frac{1}{J_k} = \frac{1}{B\omega^{1/2}} + \frac{1}{J_k} \quad (3)$$

$$B = 0.62nFC_0(D_0)^{2/3}\upsilon^{-1/6} \quad (4)$$

$$J_k = nFkC_0 \quad (5)$$

Where J is the actual measured current density, J_L means the diffusion limiting current density, J_K represents the kinetic current density. ω, n and F are angular rate of rotation, electron transfer number and the Faraday constant (F = 96485 C mol^{-1}), respectively. C_0 is the bulk concentration of O_2 with a value of 0.0012 mol L^{-1}, D_0 is the diffusion coefficient of O_2 in electrolyte with a value of 0.0000193 cm^2 s^{-1}, ν is the kinetic viscosity of KOH media (ν= 0.01 cm^2 s^{-1}), k is electron transfer rate constant. B can be gained from the slope of the K-L linear fitting. Thus, n is calculated from equation (3) when B is uncovered, and n can be obtained finally.

Rotating ring disk electrode (RRDE) measurement: The polarization curves were recorded at a rotational speed of 1600 rpm with a rate of 10 mV s^{-1}. The disk current density (I_D) represents the overall ORR catalytic activity, while the ring current density (I_R) suggests H_2O_2 production. The collection efficiency of ring electrode (N = 0.37) was pre-calibrated using standard samples.[5]

The yield of H_2O_2 production and electron transfer number (n) were calculated based on the following formulas:[6]

$$H_2O_2(\%) = \frac{200\,I_r}{N*I_d + I_r} \quad (6)$$

$$n = \frac{4I_d}{I_d + I_r/N} \quad (7)$$

The oxygen evolution reaction (OER): The OER catalytic activity of the electrocatalysts were tested *via* LSV curves were obtained at the second sweep in the range of 1.2 to 2.0 V (*vs*. RHE) at a scan rate of 10 mV s^{-1} in 1 M of KOH electrolyte, and corrected by iR compensation. The E_{onset} for OER was defined as the critical potential while the current density is 0.5 mA cm^{-2}. Converted to an RHE accorded to test correction, the overpotential (η) was calculated according to the formula η (V) = E_{RHE} − 1.23.

In situ attenuated total reflectance surface-enhanced infrared absorption spectroscopy (ATR-SEIRAS): ATR-SEIRAS spectra were measured using Bruker VERTEX 70v infrared spectrometer equipped with Shanghai Yuanfang

electrochemical in-situ cell and in-situ infrared light path accessory device (SPECEL-1). The detector is cooled with liquid nitrogen and the before testing. The catalysts were coated on the gold-coated Si crystal and put into a in O_2-saturated electrolyte. The testing was measured at different voltages, in which the collection resolution was 4 cm^{-1}, and the cumulative number of interferograms was collected for each sample spectrum is 64 scans.

Electrochemical behavior of zinc (Zn)-air battery: A polypropylene membrane (Celgard 5550) was used as the separator to separate two compartments, and a carbon cloth with 1 mg cm^{-2} of catalyst ink loaded was employed as air electrode. The Zn plate was mechanically polished and applied as the anode. The air electrode and Zn plate were assembled into a primary battery with the cathode exposed to oxygen bubbles, and 6 M KOH containing 0.2 M Zn $(OAc)_2$ was used as electrolyte. The discharge performance of the Zn-air battery was carried out using cell testing systems (Wuhan, LANDAN).

Supplementary Figures and Tables

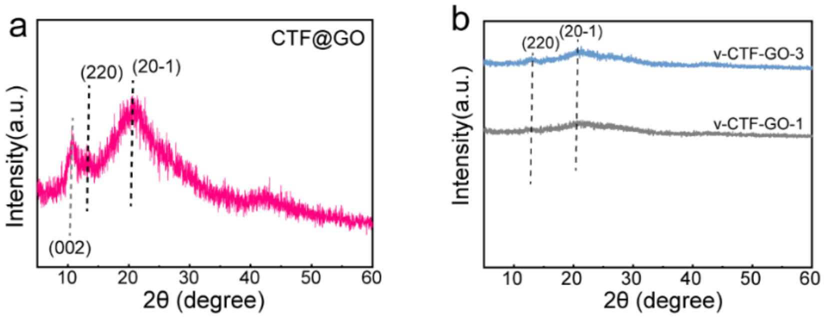

Figure S1. XRD patterns of (a) CTF@GO, and (b) v-CTF-GO-1 and v-CTF-GO-3.

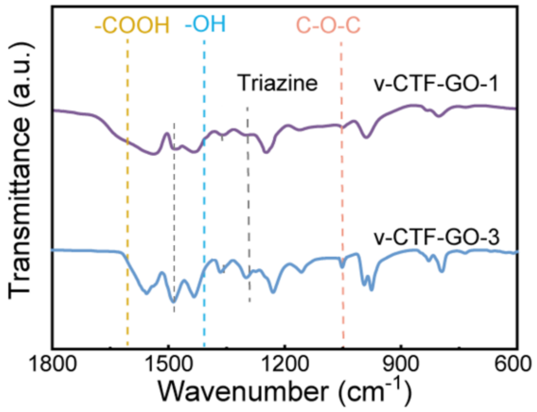

Figure S2. FT-IR spectra of and v-CTF-GO-1 and v-CTF-GO-3.

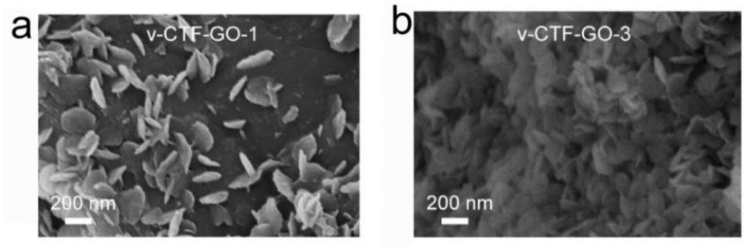

Figure S3. SEM images of (a) v-CTF-GO-1 and (b) v-CTF-GO-3.

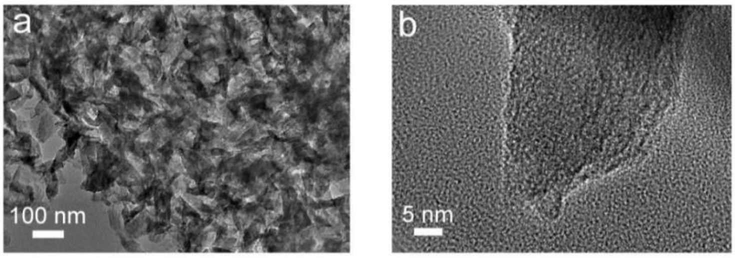

Figure S4. (a) TEM and (b) HR-TEM images of v-CTF-GO.

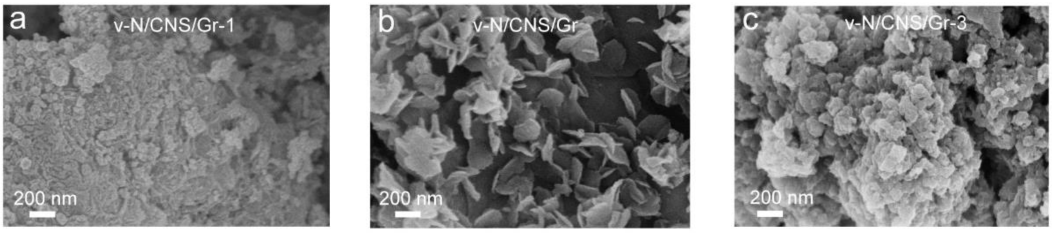

Figure S5. SEM images of (a) v-N/CNS/Gr-1, (b) v-N/CNS/Gr and (c) v-N/CNS/Gr-3.

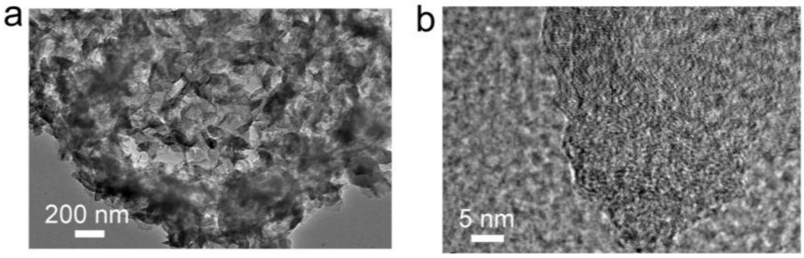

Figure S6. (a) TEM and (b) HR-TEM images of v-N/CNS/Gr.

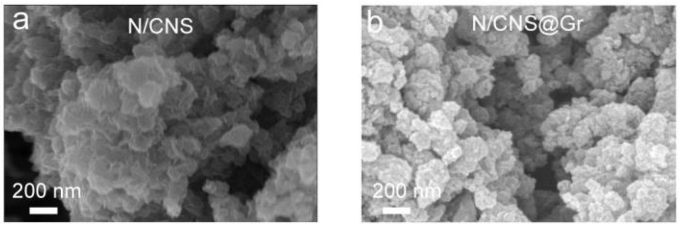

Figure S7. SEM images of (a) N/CNS and (b) N/CNS@Gr.

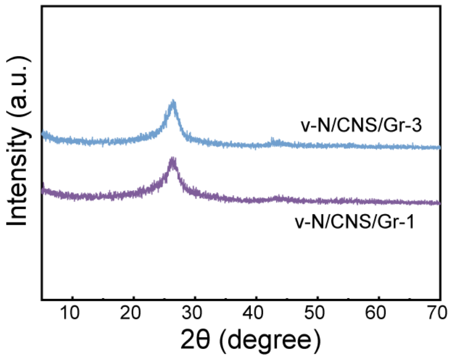

Figure S8. XRD patterns of the v-N/CNS/Gr-1 and v-N/CNS/Gr-3 catalysts.

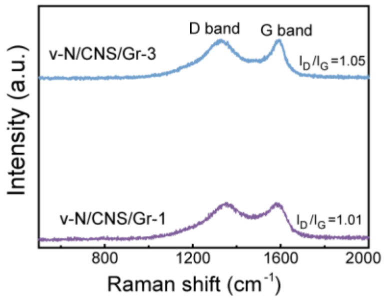

Figure S9. Raman spectra of the v-N/CNS/Gr-1 and v-N/CNS/Gr-3 catalysts.

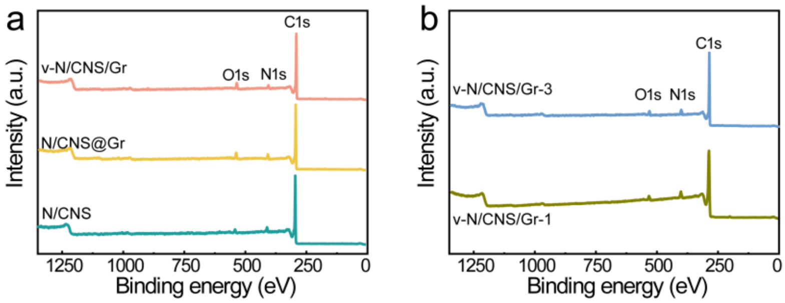

Figure S10. Full-survey XPS spectra of the as-prepared catalysts.

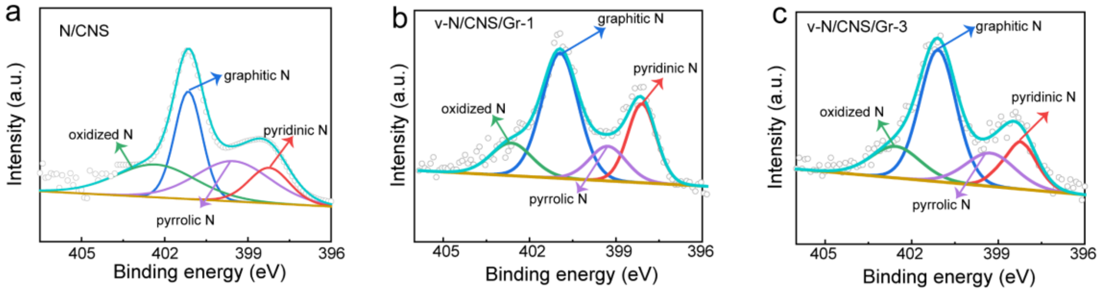

Figure S11. XPS spectra of high-resolution N 1*s* for (a) N/CNS, (b) v-N/CNS/Gr-1 and (c) v-N/CNS/Gr-3.

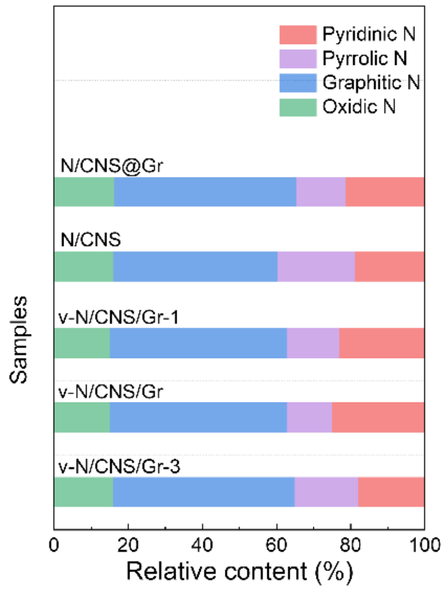

Figure S12. The nitrogen species relative content of the obtained catalysts.

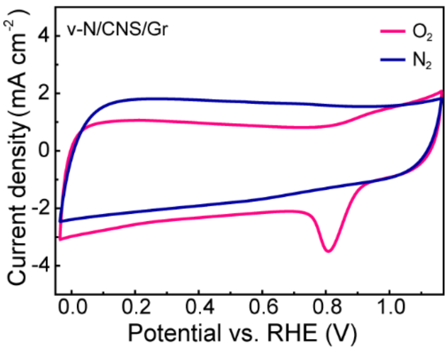

Figure S13. The CV curves of the v-N/CNS/Gr catalysts in O_2 and N_2-saturated KOH conditions.

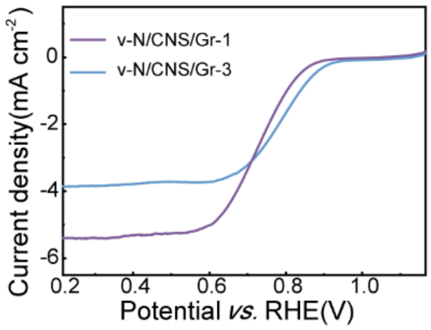

Figure S14. LSV curves of the as-obtained v-N/CNS/Gr-1 and v-N/CNS/Gr-3 electrocatalysts in 0.1 M O_2-saturated KOH.

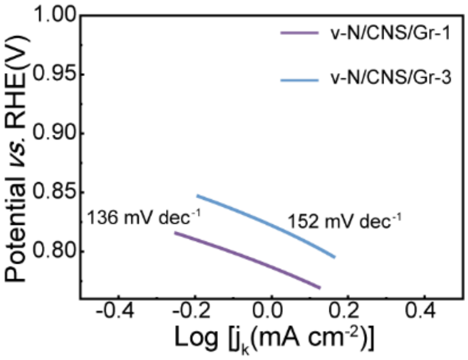

Figure S15. Tafel plot of the as-obtained v-N/CNS/Gr-1 and v-N/CNS/Gr-3 electrocatalysts in 0.1 M O_2-saturated KOH.

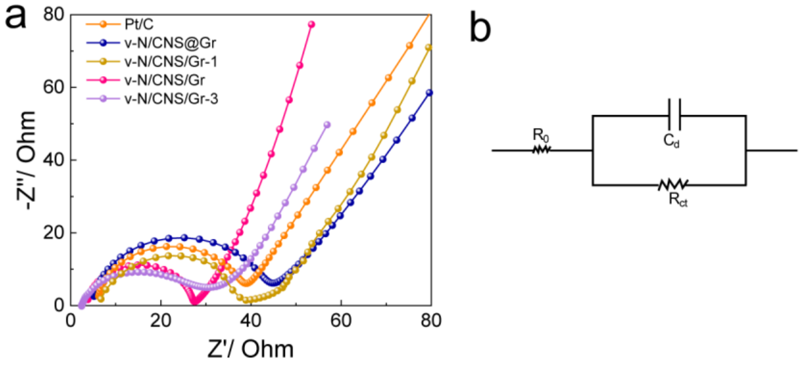

Figure S16. (a) EIS measurements of N/CNS@Gr, v-N/CNS/Gr-1, v-N/CNS/Gr, v-N/CNS/Gr-3, and Pt/C were conducted at an alternating current voltage amplitude of 5 mV with a frequency ranging from 0.01 to 100 kHz in 0.1 M KOH aqueous solution. (b) the corresponding equivalent circuit of (a).

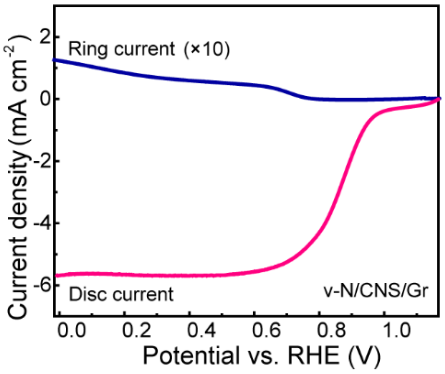

Figure S17. LSV curves of v-N/CNS/Gr detected by RRDE.

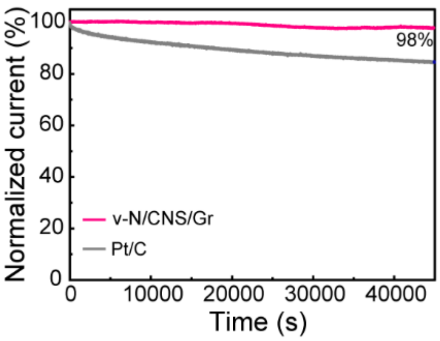

Figure S18. Relative retention $i-t$ curves of the v-N/CNS/Gr and Pt/C.

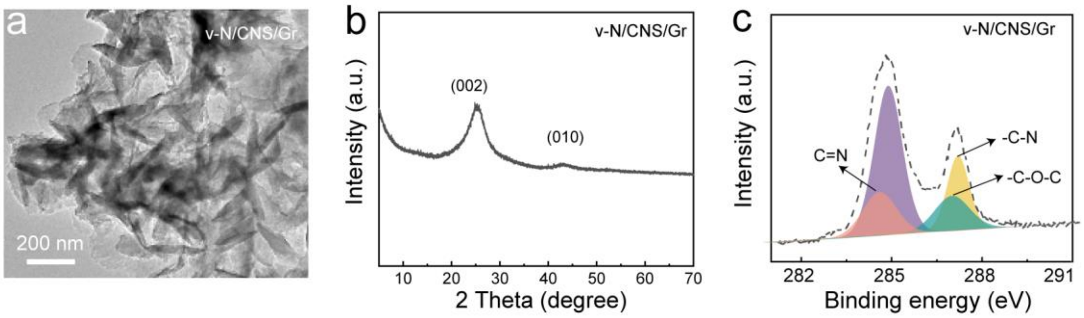

Figure S19. (a) TEM image (b) XRD pattern, and (c) high-resolution XPS C 1*s* spectrum of v-N/CNS/Gr after stability testing.

.

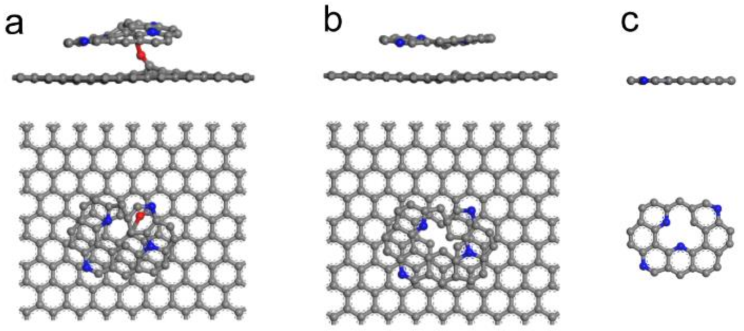

Figure S20. Different simulation models based on different catalysts of (a) v-N/CNS/Gr, (b) N/CNS@Gr and (c) N/CNS (C, N, and O atoms are shown in gray, blue, and red).

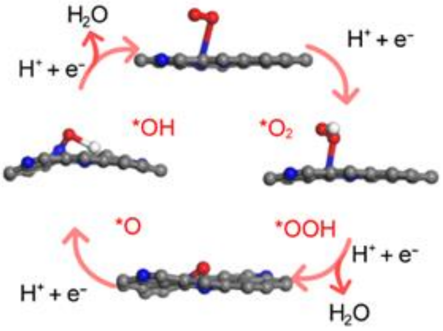

Figure S21. The proposed ORR mechanism for N/CNS.

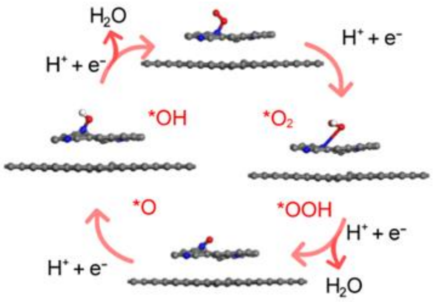

Figure S22. The proposed ORR mechanism for N/CNS@Gr.

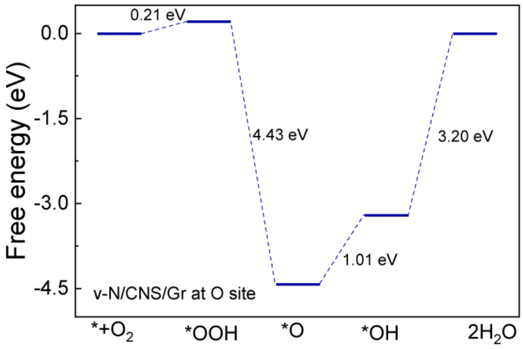

Figure S23. Free energy diagrams of ORR pathway on v-N/CNS/Gr at O site.

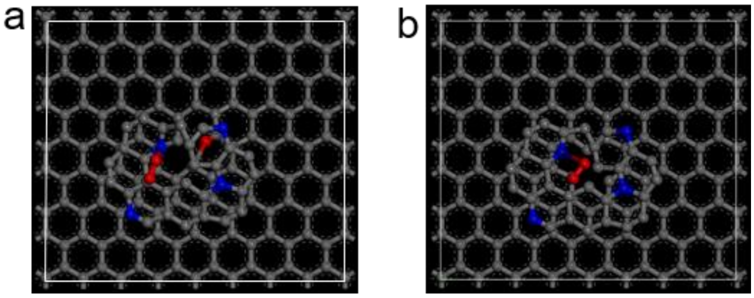

Figure S24. Millikan charge of (a) v-N/CNS/Gr and (b) N/CNS@Gr.

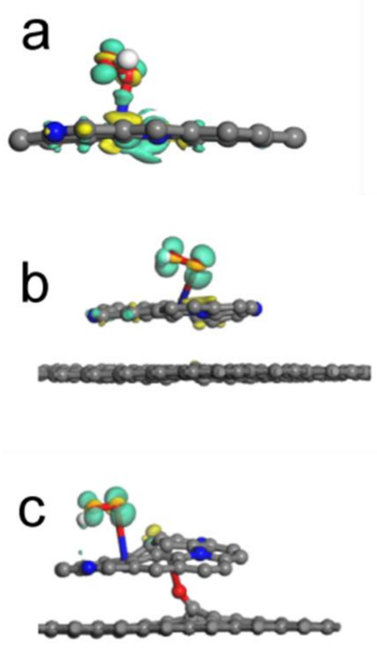

Figure S25. Electron density difference maps of *OOH adsorbed on (a) N/CNS, (b) N/CNS@Gr and (c) v-N/CNS/Gr. The yellow and blue regions are regions with increased and decreased electron density, respectively.

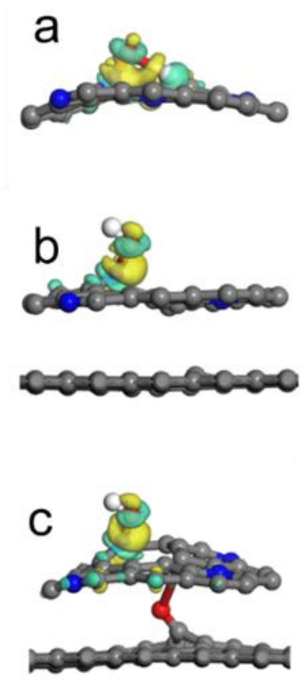

Figure S26. Electron density difference maps of *OH adsorbed on (a) N/CNS, (b) N/CNS@Gr and (c) v-N/CNS/Gr. The yellow and blue regions are regions with increased and decreased electron density, respectively.

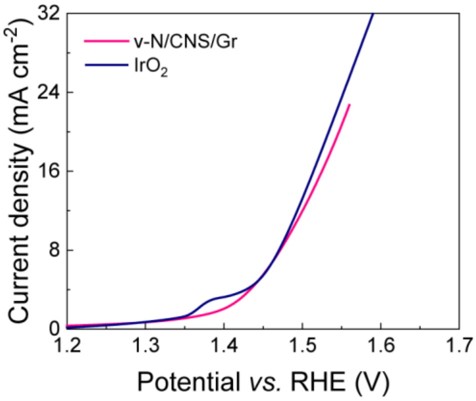

Figure S27. LSV profiles of the OER catalytic performance for v-N/CNS/Gr and IrO_2 at a scan rate of 10 mV s^{-1} in 1 M KOH electrolyte.

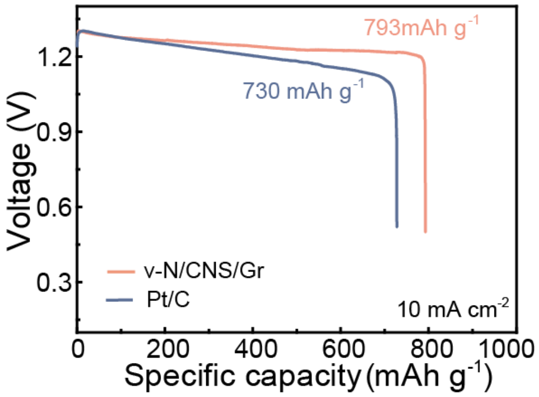

Figure S28. Specific discharge capacity at 10 mA cm^{-2} of the Zn-air battery using v-N/CNS/Gr and Pt/C electrodes.

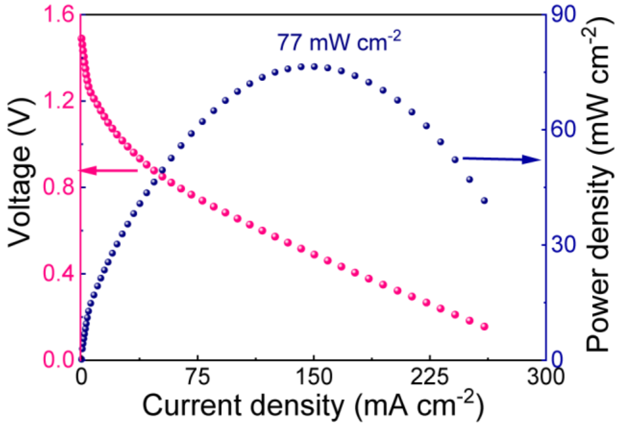

Figure S29. Discharge polarization curves and corresponding power density of the Zn-air batteries using N/CNS@Gr.

Table S1. The specific contents of the different elements in as-obtained catalysts via XPS.

Sample	C 1*s* (%)	N 1*s* (%)	O 1*s* (%)
N/CNS	94.27	4.46	1.27
N/CNS@Gr	92.79	6.05	1.16
v-N/CNS/Gr-1	93.16	5.61	1.23
v-N/CNS/Gr	92.54	6.32	1.14
v-N/CNS/Gr-3	92.48	6.19	1.33

Table S2. Summary of relative ratios of four nitrogen types in the as-prepared catalysts.

Sample	Oxidized N (%)	Graphitic N (%)	Pyrrolic N (%)	Pyridinic N (%)
N/CNS	16.1	44.2	20.9	18.8
N/CNS@Gr	16.2	49.2	13.2	21.4
v-N/CNS/Gr-1	15.3	48.1	13.7	22.9
v-N/CNS/Gr	15.5	48.3	11.5	24.7
v-N/CNS/Gr-3	16.2	49.7	16.9	17.2

Table S3. Comparisons of the specific surface area, pore volume and nitrogen contents for the N/CNS, N/CNS@Gr, v-N/CNS/Gr-1, v-N/CNS/Gr and v-N/CNS/Gr-3 electrocatalysts.

Sample	BET [m^2 g^{-1}]	Micropore volume [mL g^{-1}]	Mesopore volume [mL g^{-1}]	Total pore volume [mL g^{-1}]
N/CNS	154.6	0.039	0.148	0.187
N/CNS@Gr	312.2	0.178	0.214	0.392
v-N/CNS/Gr-1	544.9	0.239	0.308	0.547
v-N/CNS/Gr	781.4	0.203	0.694	0.897
v-N/CNS/Gr-3	300.6	0.154	0.213	0.367

Table S4. Electrical conductivity of the as-prepared catalysts measured by 4-probe method.

Sample	Conductivity (k) (S/m)	Resistivity (q) (ohm m)
N/CNS	20.7	4.8 E-02
N/CNS@Gr	31.4	3.1 E-02
v-N/CNS/Gr-1	53.1	1.8 E-02
v-N/CNS/Gr	105.6	9.4 E-03
v-N/CNS/Gr-3	72.5	1.3 E-02

The conductivity of the as-prepared catalysts was tested using a 4-probe (RTS-8).

Table S5. Comparison of the ORR catalytic performance of the v-N/CNS/Gr and metal-free heteroatom-doped carbon-based catalysts in other literatures.

Sample	Half-wave potential (V)	Onset potential (V)	Electron transfer number	Refs.
**v-N/CNS/Gr**	**0.85**	**0.96**	**3.90-3.95**	**This work**
N/S-CNH-900	0.86	1.08	~4.00	7
N/P-CNP-900	0.82	0.9	3.85	8
GLC-11	0.8	0.85	3.30	9
N, S-PGN-800	0.85	1.01	3.9-4.0	10
N/S-2DPC-60	0.86	0.90	~3.70	11
NSC-PT-0.1	0.83	0.93	-	12
VP/CNs	0.86	1.08	~4.0	13
oxazole-COF	0.75	0.85	3.7-3.9	14
NSHOPC	0.89	0.99	3.94	15
N, S-HC	0.76	0.93	3.78	16
NDPC-1000	0.84	0.97	3.8-4.0	17
NPS-HPCNFs	0.86	0.96	~4.0	18
GC	0.9	1.02	~4.0	19
Belt2	0.84	0.97	~4.0	20

Table S6. Comparison of our work with representative works on Zn-air batteries reported in other literatures.

Sample	Max power density ($mW\ cm^{-2}$)	Lifespan (h)@current density ($mA\ cm^{-2}$)	References
**v-N/CNS/Gr**	**265.3**	850 at 5 $mA\ cm^{-2}$	**This work**
SbN_4Cl/NC	193	200	*J. Am. Chem. Soc.* **2025**, *147*, 21231
Zn-N-P/NPC	252.5	400 at 20 $mA\ cm^{-2}$	*Adv. Mater.* **2025**, *37*, 2503254
La-Cl SAs/NHPC	260.7	230 at 2 $mA\ cm^{-2}$	*Adv. Mater.* **2025**, *37*, 2416387
Co-Se/Co/NC	230.2	200	*Adv. Mater.* **2025**, *37*, 2416126
$MesoCoN_3O$	185	270 at 10 $mA\ cm^{-2}$	*Adv. Mater.* **2025**, 2500370
Ir_{SA}-MnO_x	130.3	700 at 2 $mA\ cm^{-2}$	*Adv. Mater.* **2025**, *37*, 2412950
FePc-Cl-CNTs	183.5	150 at 5 $mA\ cm^{-2}$	*Angew. Chem. Int. Ed.* **2025**, *64*, e202504923
Fe_2/Ni–N–HCMs	238.3	650 at 5 $mA\ cm^{-2}$	*Angew. Chem. Int. Ed.* **2025**, *64*, e202421168
RCOF	194	/	*Angew. Chem. Int. Ed.* **2025**, *64*, e202424449
CoCo-BiSalphen@KB	216	500 at 5 $mA\ cm^{-2}$	*Nat. Commun.* **2025**, *16*, 921
A-MnO_2/NSPC	181	287 at 5 $mA\ cm^{-2}$	*Adv. Mater.* **2024**, *36*, 2312868.
$Fe_{SA/AC}$@HNC +RuO_2	172	131 at 5 $mA\ cm^{-2}$	*Adv. Mater.* **2024**, *36*, 2400523
Ni/$MnFe_2O_4$	120	360 at 8 $mA\ cm^{-2}$	*Adv. Mater.* **2024**, *36*, 2400572
Fe/I-N-CR	197.9	280 at 20 $mA\ cm^{-2}$	*Adv. Mater.* **2024**, *36*, 2412978
FeCo-N_3O_3@C	143	200 at 10 $mA\ cm^{-2}$	*Nat. Synth.* **2024**, 3, 878
CR-Co/ClNC	176.6	/	*Nat. Commun.* **2024**, 15, 1675.
T-Fe SAC	199	350 at 20 $mA\ cm^{-2}$	*Angew. Chem. Int. Ed.* **2024**, 63, e202319370
FeMn-N-C	151	700 at 1 $mA\ cm^{-2}$	*Adv. Mater.* **2024**, 36, 2405763
FeSn-C_2N	265.5	350 at 10 $mA\ cm^{-2}$	*J. Am. Chem. Soc.* **2024**, *146*, 21357
YN_4-Cl	162	/	*Adv. Mater.* **2023**, 35, e2300381
FeNi LDH-TpF6-2	118	/	*Adv. Mater.* **2023**, 2210550
Fe SAs HS	170	70 at 20 $mA\ cm^{-2}$	*Angew. Chem. Int. Ed.* **2023**, 62, 202304229
P/Fe–N–C	269	192 at 10 $mA\ cm^{-2}$	*J. Am. Chem. Soc.* **2023**, 145, 3647
(Fe,Ni)Se_2	215	350 at 20 $mA\ cm^{-2}$	*J. Am. Chem. Soc.* **2023**, 145, 20248

REFERENCES

(1) Hummers, W. S. Jr.; Offeman, R. E. Preparation of graphitic oxide. *J. Am. Chem. Soc.* **1958**, *80*, 1339-1339.

(2) Jiang, D.; Cao, X.; Shi, Y.; Chen, J.; Li, X.; Liu, J.; Zhou, H. Flexible Ti_3C_2Tx MXene regulated photoelectrochemical sensing platform for sensitive monitoring of dopamine. *Adv. Funct. Mater.* **2024**, *34*, 2410546.

(3) Cao, X.; Zhao, L.; Wulan, B.; Tan, D.; Chen, Q.; Ma, J.; Zhang, J. Atomic bridging structure of nickel–nitrogen–carbon for highly eficient electrocatalytic reduction of CO_2. *Angew. Chem. Int. Ed.* **2022**, *61*, e202113918.

(4) Wang, J.; Hu, C.; Wang, L.; Yuan, Y.; Zhu, K.; Zhang, Q.; Yang, L.; Lu, J.; Bai, Z. Suppressing thermal migration by fine-tuned metal-support interaction of iron single-atom catalyst for efficient ORR. *Adv. Funct. Mater.* **2023**, *33*, 2304277.

(5) Su, Y.; Yao, Z.; Zhang, F.; Wang, H.; Mics, Z.; Cánovas, E.; Bonn, M.; Zhuang, X.; Feng, X. Sulfur-enriched conjugated polymer nanosheet derived sulfur and nitrogen co-doped porous carbon nanosheets as electrocatalysts for oxygen reduction reaction and zinc–air battery. *Adv. Funct. Mater.* **2016**, *26*, 5893-5902.

(6) Lu, T.; Sun, M.; Wang, F.; Chen, S.; Li, Y.; Chen, J.; Liao, X.; Sun, X.; Liu, Y.; Wang, F.; Wang, H. Selective oxidation of sp-bonded carbon in graphdiyne/carbon nanotubes heterostructures to form dominant epoxy groups for two-electron oxygen reduction. *ACS Nano* **2024**, *18*, 15035-15045.

(7) Chen, X.; Guan, J.; Zheng, Y.; Shen, Y.; Chen, R.; Huang, N.; Jia, B.; Kong, X. Y.; Yan, Y.; Liu, M.; Ye, L. Self-assembled covalent triazine frameworks derived N, S co-doped carbon nanoholes with facilitating ions transportation toward remarkably enhanced oxygen reduction reaction and for zinc–air batteries. *Small* **2025**, *21*, 2410619.

(8) Ding, R.; Zhang, D.; Bi, L.; Shi, S.; Tang, X.; Zhang, Z.; He, Y. N/P-codoped carbon nanotubes for efficient oxygen reduction reaction. *ACS Appl. Nano Mater.* **2023**, *6*, 21887-21896.

(9) Ye, X.-w.; Hu, L.-b.; Liu, M.-c.; Wang, G.; Yu, F. Improved oxygen reduction performance of a N, S co-doped graphene-like carbon prepared by a simple carbon bath method. *New Carbon Mater.* **2020**, *35*, 531-539.

(10) Qian, Q.; Hu, H.; Huang, S.; Li, Y.; Lin, L.; Duan, F.; Zhu, H.; Du, M.; Lu, S. Versatile hyper-cross-linked polymer derived porous carbon nanotubes with tailored selectivity for oxygen reduction reaction. *Carbon* **2023**, *202*, 81-89.

(11) Li, N.; Li, M.; Guo, K.; Guo, Z.; Wang, R.; Bao, L.; Hou, G.-L.; Lu, X. Deciphering the role of native defects in dopant-mediated defect engineering of carbon electrocatalysts. *Adv. Energy Mater.* **2024**, *14*, 2401008.

(12) Wang, S.; Chen, Y.; Zhao, Y.; Wei, G.; Li, D.; Liu, X. Mesopore-dominated N, S co-doped carbon as advanced oxygen reduction reaction electrocatalysts for Zn-air battery. *J. Mater. Sci.* **2022**, *57*, 19431-19446.

(13) Xia, H.; Pang, R.; Dong, X.; Liu, Q.; Chen, J.; Wang, E.; Li, J. Boosting oxygen reduction reaction kinetics by designing rich vacancy coupling pentagons in the defective carbon. *J. Am. Chem. Soc.* **2023**, *145*, 25695-25704.

(14) Li, X.; Yang, S.; Liu, M.; Yang, X.; Xu, Q.; Zeng, G.; Jiang, Z. Catalytic linkage engineering of covalent organic frameworks for the oxygen reduction reaction. *Angew. Chem. Int. Ed.* **2023**, *62*, e202304356.
(15) Han, H.; Guo, Y.; Wang, X.; Zhang, X. In-situ gas foaming synthesis of N, S-rich co-doped hierarchically ordered porous carbon as an efficient oxygen reduction reaction catalyst. *J. Colloid Interface Sci.* **2023**, *646*, 167-175.
(16) Wang, M. J.; Wang, L.; Li, Q.; Bai, Y.; Wang, D.; Feng, Y. Controllable synthesis of N-/S-doped and N,S-doped hollow carbon spheres for the oxygen reduction reaction: A universal mono-micelle self-assembly strategy. *Mater. Lett.* **2022**, *309*, 131315.
(17) Yang, L.; Liu, H.; Qiao, Z.; Sun, P.; Li, D.; Jiang, R.; Liu, S.; Niu, Z.; Zhang, Y.; Lin, T.; Zhang, Q.; Gu, L.; Wang, S.; Cao, D.; Chen, Z. Highly active and durable metal-free carbon catalysts for anion-exchange membrane fuel cells. *Adv. Energy Mater.* **2023**, *13*, 2204390.
(18) Xu, X.; Wu, H.; Yan, Y.; Zheng, Y.; Yan, Y.; Qiu, S.; Liang, T.; Deng, C.; Yao, Y.; Zou, J.; Liu, M. Electronic structure modulation in N, P, S tri-doped nanofibers with interpenetrated pores for enhanced oxygen reduction reaction. *Adv. Energy Mater.* **2025**, *15*, 2405236.
(19) Zhai, Z.; Wang, Y.-J.; Pan, L.; Huang, F.; Liu, D.; Wang, B. Accelerating O-O bond dissociation in oxygen reduction reaction on the sp^3-hybridized carbon. *Appl. Surf. Sci.* **2025**, *691*, 162668.
(20) Xu, T.; Zhang, X.; Wang, Z.; Ng, P. W.; Jiao, L.; Wang, S.-Q.; Khoo, K. H.; Xu, Z.; Wu, J.; Zhu, J. Modulating the cavity size of carbon nanobelts for enhanced oxygen reduction reaction. *ACS Appl. Mater. Interfaces* **2025**, *17*, 20096-20104.
